# A Conserved Sequence Extending Motif III of the Motor Domain in the Snf2-Family DNA Translocase Rad54 Is Critical for ATPase Activity

**DOI:** 10.1371/journal.pone.0082184

**Published:** 2013-12-16

**Authors:** Xiao-Ping Zhang, Ryan Janke, James Kingsley, Jerry Luo, Clare Fasching, Kirk T. Ehmsen, Wolf-Dietrich Heyer

**Affiliations:** 1 Department of Microbiology and Molecular Genetics, University of California Davis, Davis, California, United States of America; 2 Department of Molecular and Cellular Biology, University of California Davis, Davis, California, United States of America; Saint Louis University, United States of America

## Abstract

Rad54 is a dsDNA-dependent ATPase that translocates on duplex DNA. Its ATPase function is essential for homologous recombination, a pathway critical for meiotic chromosome segregation, repair of complex DNA damage, and recovery of stalled or broken replication forks. In recombination, Rad54 cooperates with Rad51 protein and is required to dissociate Rad51 from heteroduplex DNA to allow access by DNA polymerases for recombination-associated DNA synthesis. Sequence analysis revealed that Rad54 contains a perfect match to the consensus PIP box sequence, a widely spread PCNA interaction motif. Indeed, Rad54 interacts directly with PCNA, but this interaction is not mediated by the Rad54 PIP box-like sequence. This sequence is located as an extension of motif III of the Rad54 motor domain and is essential for full Rad54 ATPase activity. Mutations in this motif render Rad54 non-functional *in vivo* and severely compromise its activities *in vitro*. Further analysis demonstrated that such mutations affect dsDNA binding, consistent with the location of this sequence motif on the surface of the cleft formed by two RecA-like domains, which likely forms the dsDNA binding site of Rad54. Our study identified a novel sequence motif critical for Rad54 function and showed that even perfect matches to the PIP box consensus may not necessarily identify PCNA interaction sites.

## Introduction

Homologous recombination (HR) is a ubiquitous DNA metabolic process that is essential for meiotic chromosome segregation, the repair of complex DNA damage such as DNA double-stranded breaks (DSB) or interstrand crosslinks, and the recovery of stalled or broken replication forks [1], [2]. HR is catalyzed by a suite of genes/proteins collectively called the *RAD52* epistasis group, and the process can be conceptually divided into three stages: presynapsis, synapsis, and postsynapsis [3]. During presynapsis, the DNA substrate is processed to allow the formation of the Rad51 nucleoprotein filament on ssDNA. The Rad51 filament catalyzes the signature reactions of HR, homology search and DNA strand invasion, which define synapsis. During postsynapsis, DNA synthesis from the 3′-end of the invading DNA end restores the missing genetic information and the resulting joint molecules are processed to either crossover or noncrossover products, where the continuity of the chromosome has been restored.

*RAD54* has been identified in the budding yeast *Saccharomyces cerevisiae* as a core member of the *RAD52* epistasis group and together with mutants in the *RAD51* and *RAD52* genes, *rad54* mutants are the most ionizing radiation-sensitive single gene mutants in budding yeast [4]. The gene is well conserved throughout evolution in eukaryotes [5], although HR is somewhat less dependent on RAD54 in vertebrates [6]. Eukaryotes also contain a close paralog of Rad54, Rdh54/Tid1 in budding yeast and RAD54B in vertebrates [7]. The existence of these paralogs in part explains the comparatively mild meiotic defect of the budding yeast *rad54* mutant relative to *rad51* or *rad52* [8], [9] and the reduced dependence of HR on RAD54 in vertebrates [10].

Rad54 is a member of the Snf2-family of dsDNA-dependent/stimulated ATPases in the SF2 family of DNA helicases/DNA translocases [11]. These motor proteins translocate on duplex DNA and remodel specific protein-dsDNA complexes. The ATP-dependent chromatin remodeling complex SWI/SNF contains the founding member of this group of proteins, Swi2/Snf2, as the catalytic core of its chromatin remodeling activity [12]. Genetic analysis has established that Rad54 functions after the assembly of the Rad51-ssDNA filament in synapsis and/or postsynapsis [13], [14]. Extensive biochemical analysis revealed that Rad54 exerts many functions during the recombination process [7], [15], [16]. While Rad54 can stabilize the Rad51-ssDNA filament in an ATPase-independent fashion [17]–[19], other functions of Rad54 require its ATPase activity, including DNA strand invasion, branch migration, chromatin remodeling, and Rad51 dissociation from heteroduplex DNA to allow access of DNA polymerases to the 3′-OH end of the invading strand [7], [15], [16], [20]. Genetic analysis established that the ATPase activity is essential for Rad54 function, and mutations in the Walker A box cause DNA damage sensitivity as extreme as the gene deletion [21], [22].

Gorbalenya and Koonin identified a large group of proteins that shared 7 sequence motifs labeled I, Ia, II, III, IV, V, and VI, which were originally proposed to specify helicase activity [23]. Motif I corresponds to the Walker A box motif (GxGKT/S), which is essential for ATP binding and hydrolysis, and motif II corresponds to the Walker B box [24]. Later it became clear that these motifs identify proteins that directionally translocate on ssDNA or dsDNA powered by ATP hydrolysis, of which DNA helicases capable of separating the two strands of a duplex DNA are only a subgroup [25]. This class of proteins is divided into two superfamilies, SF1 and SF2. Each superfamily can be subdivided into many families, including the Snf2 family within the SF2 superfamily, which itself can be further divided into distinct Snf2 subfamilies on the basis of additional conserved sequence motifs. The Rad54-like subfamily contains Rad54 and its paralogs Rdh54/Tid1 and RAD54B [11]. The functions of individual subfamily-specific motifs are not understood [11].

**Figure 1:**
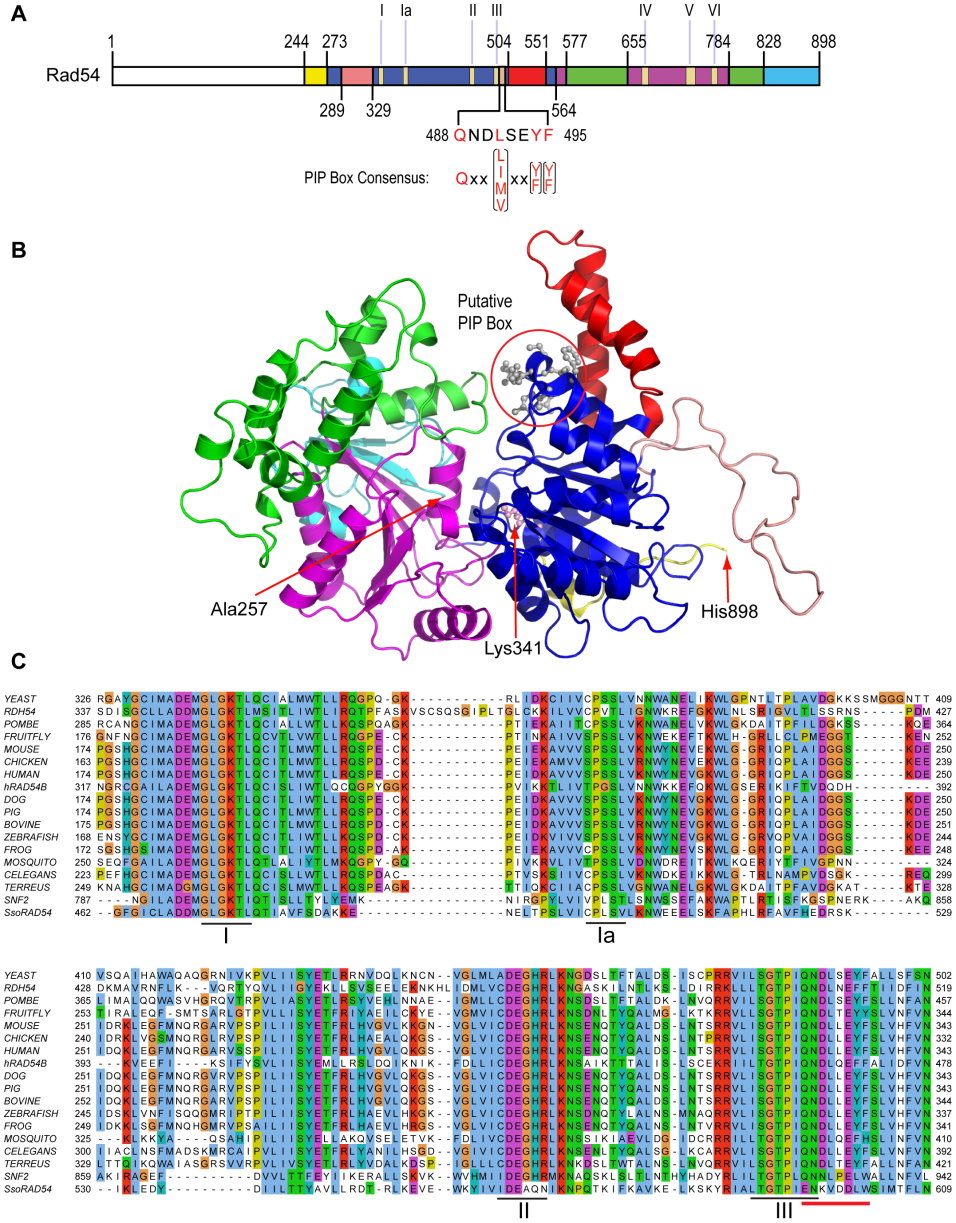
*Saccharomyces cerevisiae* Rad54 and its homologs. **A**. Schematic representation of the budding yeast Rad54 protein sequence. The color scheme relates to (**B**). The motifs of the motor domain are labeled I–VI [11], [47]. The PIP box motif (amino acids 488–495) is shown in alignment with the PIP box consensus sequence [26], [27]. **B**. *S. cerevisiae* Rad54 structural model. The model was generated based on the zebrafish Rad54 crystal structure (1Z3I.pdb) [47]. The putative PIP box is represented in ball-sticks mode (circled). Rad54 termini (N-terminal Ala257, C-terminal His898) and the catalytic residue of the Walker A box (Lys341) are shown. **C**. Sequence comparison of Rad54 and its paralogs. Only sequence regions encompassing motifs I, Ia, II and III are shown. The PIP box-like sequence is marked by a red line and extends motif III. The Rad54 and Rad54-like sequences were retrieved from Uniprot database (UniProt release 2011_12–Dec 14, 2011): YEAST, *Saccharomyces cerevisiae*, P32863; RDH54, *S. cerevisiae*, P38086; POMBE, *Schizosaccharomyces pombe*, P41410; FRUIT FLY, *Drosophila melanogaster*, O76460; MOUSE, *Mus musculus*, P70270; CHICKEN, *Gallus gallus*, O12944; HUMAN, *Homo sapiens*, Q92698; hRAD54B, *H. sapiens*, Q9Y620; DOG, *Canis familiaris*, F1PJC9; PIG, *Sus scrofa*, B0M1M8; BOVINE, *Bos taurus*, A1L4Z4; ZEBRAFISH, *Danio rerio*, Q7ZV09; FROG, *Xenopus tropicalis*, Q6NVL9; MOSQUITO, *Culex quinquefasciatus*, B0W5L6; CELEGANS, *Caenorhabditis elegans*, G5EEN6; TERREUS, *Aspergillus terreus*, Q0CSL2; SNF2, *S. cerevisiae*, P22082; SsoRAD54, *Sulfolobus solfataricus*, Q97XQ7. The alignment is colored by a ClustalX color scheme that integrates amino acid properties and evolutionary conservation.

Proliferating Nuclear Cell Antigen (PCNA) is the sliding clamp in eukaryotes that imparts processivity to DNA polymerases [26]. PCNA also interacts with a host of other proteins that act on DNA and chromatin [26]. The interaction with PCNA is often mediated by a specific PCNA interaction motif called the PIP box [27] (see Fig. 1A), which is often found on a flexible tether on the extreme N- or C-terminus of the PCNA-interacting protein [26]. An alternative PCNA interaction motif, APIM, has also been defined in many proteins, sometimes in conjunction with a PIP box [28]. The trimeric nature of eukaryotic PCNA affords the sliding clamp the potential to interact with up to three binding partners.

We identified a perfect match to the PIP box consensus sequence in the Rad54 protein sequence and found that Rad54 physically interacts with PCNA. Given the role of Rad54 in an HR step directly preceding PCNA loading by RFC [29], [30], we speculated whether the Rad54 PIP box has functional significance in the transition from DNA strand invasion to D-loop extension by DNA synthesis. However, we found that the Rad54-PCNA interaction is independent of the putative PIP box of Rad54. Instead this sequence motif is an extension of the ATPase motif III, specific for the RAD54 subfamily of Snf2 family translocases. Mutations in this extended motif affect the Rad54 ATPase activity and dsDNA binding, compromising its role in Rad51-mediated D-loop formation. Furthermore, mutations in this sequence motif abolish Rad54 function *in vivo* and lead to DNA damage sensitivity comparable to that of the gene deletion.

## Materials and Methods

### Media and strains

Complete YPD and SD-ura media (Synthetic minimal (SD), 0.67% yeast nitrogen base without amino acids, 2% dextrose, 0.87 g/liter SD-Ura amino acid drop-out mixture) were used for yeast growth. For Rad54 overexpression, basic yeast media (0.67% yeast nitrogen base without amino acids, 2% (w/v) sodium lactate, 3% (v/v) glycerol and 0.87 g/liter SD-ura amino acid drop-out mixture was used. Rad54 wild type protein was overexpressed in WDHY668, the mutant protein in WDHY2655 (*rad54*-Δ). Native *RAD54* was replaced with a *rad54-Y494A,F495A* (abbreviated as *rad54*-2A) integration cassette generated by cloning-free adaptamer-mediated PCR as described [31]. Briefly, pWDH767 and pWDH729 were used as PCR templates to generate integration cassettes. A single candidate was confirmed by sequencing of the entire *RAD54* locus and archived as WDHY2625. The strains used in this study are listed in Table S1 In File S1.

### Proteins

Yeast PCNA, RFC and Pol δ used for D-loop extension assay were gifts from Peter Burgers. The yeast PCNA used for protein-protein interaction assays was purified through G T-affinity chromatography. The PCNA gene was subcloned from plasmid pWDH716 (from *E. coli* RDK4511, a gift from Richard Kolodner) into a modified pET14b vector that adds a GST-tag on the N-terminus of PCNA. GST-PCNA was overexpressed in *E. col* strain BL21(DE3) and purified with glutathione sepharose 4B (Pharmacia). The GST-tag was cleaved on the column at 4°C using PreScission protease overnight. After cleavage, four residues were left on the PCNA N-terminus, GPRD. Yeast Rad51, Rad54 and RPA were purified as previously described [32]. The “PCNA bacterial extract” was from RDK4511, which expresses PCNA without IPTG induction, in which the PCNA accounts for ∼40–50% total protein from the extract (data not shown).

### Oligonucleotides

The sequence for the oligonucleotides used for cloning, *in vitro* mutagenesis, and genomic manipulations are listed in Table S2 in File S1.

### Sequence analysis

Rad54 and its homologs/paralogs were retrieved from UniProt database release 2011_12–Dec 14, 2011. The sequences were aligned with MUSCLE 3.0 [33] in Jalview 2.7 [34]. The alignment was colored by a ClustalX [35] color scheme that integrated properties of amino acids and their conservation. A partial alignment including helicase domain I through the extended domain III is presented in Figure 1C.

### Rad54 structure modeling

The *S. cerevisiae* Rad54 sequence was submitted to BioInfoBank Meta Server (http://meta.bioinfo.pl/submit_wizard.pl). As expected, the zebrafish Rad54 X-ray crystal structure (pdb code: 1Z3I) was recognized as the best homology template. These two proteins share 38.8% identity and 49.73% similarity. The alignment generated by the Meta Server was confirmed by ClustalW [35] and MUSCLE [33] in Jalview [34]. 1,000 Rad54 structural models were generated in Modeller 9v10 and the 100^th^ model was identified as the best candidate based on the PDF score. Two loops, residues 397–406 and 800–812, form part of the surface of the cleft formed by the N- and C-terminal RecA domains, which were refined through the loop-modeling protocol of Modeller 9v10 [36]. 10,000 models were generated during refinement and the 3,405^th^ model was selected as the final candidate and was validated as described in [37]. The model was visualized in PyMol v1.5 (http://pymol.org/) (Figure 1B) and used for dsDNA fragment docking analysis (Fig. S2 in File S1).

### In vitro Rad54 and PCNA interaction

The purified GST-Rad54 and PCNA or PCNA bacterial extract were incubated in 100 µl (final volume) PCNA interaction buffer containing 25 mM Tris-HCl, pH 7.5, 50 mM NaCl, 1 mM DTT, 10% glycerol and 0.05% NP-40 at room temperature for 1 hr with agitation. Following pre-treatment with BSA, 20 µl of 50% glutathione sepharose beads (Pharmacia) in interaction buffer was added, and the reaction was incubated with agitation for one hr. Then the mixture was centrifuged at 500×g for five min and the supernatant completely removed. The beads were washed with 250 µl ice cold wash buffer (interaction buffer with 100 mM NaCl) three times, removing the buffer by centrifugation at 4°C. Finally, 20 µl 1× Laemmli buffer was added, and the sample was boiled at 95°C for 3 minutes. The supernatant was electrophoresed on a 12% SDS-PAGE gel, which was either stained with Coomassie blue directly or was transferred to a nitrocellulose membrane and detected with yeast PCNA and Rad54 antibodies using the standard ECL method [38].

### Mutagenesis

Mutagenesis was performed according to the Stratagene (Agilent Technologies) QuikChange site-directed mutagenesis protocol using the wild-type yeast Rad54 plasmid pWDH597 as a template. Primers were synthesized by Operon (http://www.operon.com; primer sequences are listed in Table S2 in File S1). The mutant plasmid was amplified in *E. coli* DH5alpha. The entire *rad54* sequence in the mutant plasmids was confirmed by DNA sequencing.

### Immunoblots

Whole cell extracts prepared from 10 OD units of cells from wild type (WDHY2217), *rad54-2A* (WDHY2625) and *rad54*-Δ (WDHY2571) strains (Table S1 in File S1) were separated on a 4–12% gradient SDS-PAGE gel (Lonza) at 12 V/cm for 1 hr. The proteins were transferred onto a nitrocellulose membrane at 300 mA for 1 hr, blocked with 5% Nonfat Dry Milk (Safeway) in 1× Phosphate Buffered Saline (PBS) and incubated with affinity-purified rabbit anti-*S. cerevisiae* Rad54 antibodies (1∶200) for 1 hr at room temperature [21]. The membrane was washed and incubated with goat anti-rabbit-HRP (DAKO; 1∶5,000) for 30 min at room temperature. The proteins were detected with ECL Plus (Amersham) and visualized on HyperFilm ECL (Amersham) for 20 min.

### D-loop extension assays

The Rad51- and Rad54-supported D-loop extension assays were performed as described [20], [30]. 20 nM ^32^P-labeled 95-mer oligonucleotide was incubated with 0.618 µM Rad51 at 30°C for 10 min in 30 mM tris-acetate buffer (pH 7.5) containing 1 mM DTT, 5 mM ATP, 20 mM magnesium acetate, 20 mM phosphocreatine, 0.1 µg/µl creatine kinase, 50 µg/ml bovine serum albumin and 75 µM dNTP. After adding 0.1 µM RPA, the incubation was continued for another 10 min. Then 5 nM supercoiled pUC19 and 60 nM Rad54 was added into the system and the mixture was incubated at 30°C for 5 minutes. At this point, 20 nM PCNA and 20 nM RFC were added and the reaction was incubated for another 5 minutes. The extension reaction was started by adding 20 nM DNA polymerase δ. Samples were withdrawn before and at 2, 5, 10 minutes after addition of the DNA polymerase. For extension assays with Klenow polymerase, the same conditions were used except that PCNA, RFC, and Pol δ were omitted and 40 nM Klenow was added instead.

### ATPase assay

The NADH-coupled ATPase assay was used as described [39] with minor modifications. Rad54 (wild-type or Rad54-2A) was diluted to a 150 nM stock in storage buffer (50 mM Tris-HCl, pH 7.5, 500 mM NaCl, 50% glycerol, 0.2 mM PMSF, 0.1 mM DTT) and the ATPase reaction was initiated by adding 1 µl enzyme to a pre-aliquoted volume of 149 µL reaction buffer (25 mM HEPES, pH 7.5, 2.5 mM Mg(OAc)_2_ (free concentration; additional Mg(OAc)_2_ was added where appropriate at 1∶1 relative to ATP concentration), 1 mM DTT, 100 µg/ml BSA, 2 mM PEP, 36 U/ml pyruvate kinase (type II from rabbit muscle; Sigma), 20 U/ml lactate dehydrogenase (Sigma), 1.5 µM pUC19 (NEB) and ATP at 0, 0.1, 0.25, 0.5, 1, 2. 2.5 or 5 mM), with NADH (Sigma; resuspended in water) added to 0.2–0.4 µg/ml immediately prior to Rad54. Reactions were mixed for 1 min on a shaker at 23°C, bringing Rad54 to a final assay concentration of 5 nM (monomer). Kinetics of NADH depletion were measured at λ = 340 nm, 30°C in a Spectramax 250 microplate spectrophotometer (Molecular Devices), sampled at 60 second intervals up to 45 min. NADH depletion was plotted in GraphPad Prism (http://www.graphpad.com/) and the slope of the initial rate (linear between 10–45 min at all ATP concentrations) was determined for three independent replicates, converted to velocity as described in [39] and plotted as a function of ATP concentration. Data were fitted to a Michaelis-Menten hyperbolic function in Prism, from which *K_M_* and *V_max_* were determined. *k_cat_* was calculated as *V_max_*/[Rad54]. Single time point Rad54 and Rad54-2A ATPase activity was analyzed using a charcoal assay [40].

### DNA binding assay

Binding of Rad54 to dsDNA was analyzed by an electrophoretic mobility shift gel assay similar to that in [36]. The reaction buffer contains 25 mM triethanolamine acetate (TEA-Ac), pH 7.5, 13 mM magnesium acetate, 1.8 mM DTT, 100 µg/ml BSA, 11.25% glycerol and 113 mM NaCl. 30 µM bp pUC19 circular dsDNA and the indicated amount of Rad54 were incubated at 30°C for 15 min in the absence of nucleotide or presence of 5 mM ATP plus ATP-regenerating system (20 mM phosphocreatine and 0.1 µg/µl creatine kinase) or 5 mM ATP-γ-S. Then the DNA-protein complexes were fixed by adding 5% glutaraldehyde (in 100 mM TEA-Ac, pH 7.5) to a final concentration of 0.25% and incubated for another 15 min. The reaction mixture was separated on 1% agarose gels in TAE at 80 V for 120 min followed by staining of 1 µg/µl ethidium bromide TAE and UV visualization on Alpha Innotech FluorChem 8900 bioimaging system. The DNA-protein complexes were quantified with ImageQuant 5.2.

## Results

### Rad54 contains a perfect match to the PIP box consensus sequence

Rad54 dissociates Rad51 from the heteroduplex product of DNA strand exchange, allowing DNA polymerases to access the 3′-end of the invading strand [29], [30]. In budding yeast, the PCNA-dependent DNA polymerase δ appears to be the primary DNA polymerase to extend the invading strand in the D-loop based on genetic and biochemical evidence [20], [41]–[46]. Rad54 amino acid sequence inspection revealed an eight-amino acid region (residues 488–495, Fig. 1A) that perfectly matches the consensus PCNA interaction motif (PIP box) [26], [27], immediately C-terminal to motif III of the Rad54 ATPase domain. Using the crystal structure of zebrafish RAD54 [47] as a template for *S. cerevisiae* Rad54, we generated a structural model and located the PIP box on the surface of the internal cleft (Fig. 1B) that likely defines the dsDNA binding site of Rad54 (see Fig. S2 in File S1) [47]. Sequence analysis of Rad54 homologs reveals that this sequence is highly conserved across eukaryotes; in fact, the key residues of the PIP box consensus motif (Qxx[LIMV]xx[YF][YF]) are perfectly conserved (Fig. 1C). Moreover, the Rad54 paralogs (Rdh54/Tid1 in budding yeast and RAD54B in humans), which have a similar, partially overlapping function to Rad54 [7], share the identical sequence motif with Rad54 (Fig. 1C). Considering the fact that Rad54 acts in recombination at the step immediately preceding PCNA loading and the initiation of recombination-associated DNA synthesis by a PCNA-dependent enzyme, we were interested in exploring the functional significance of this potential PCNA interaction site.

### Rad54 interacts with PCNA independent of a functional PIP box consensus sequence

**Figure 2:**
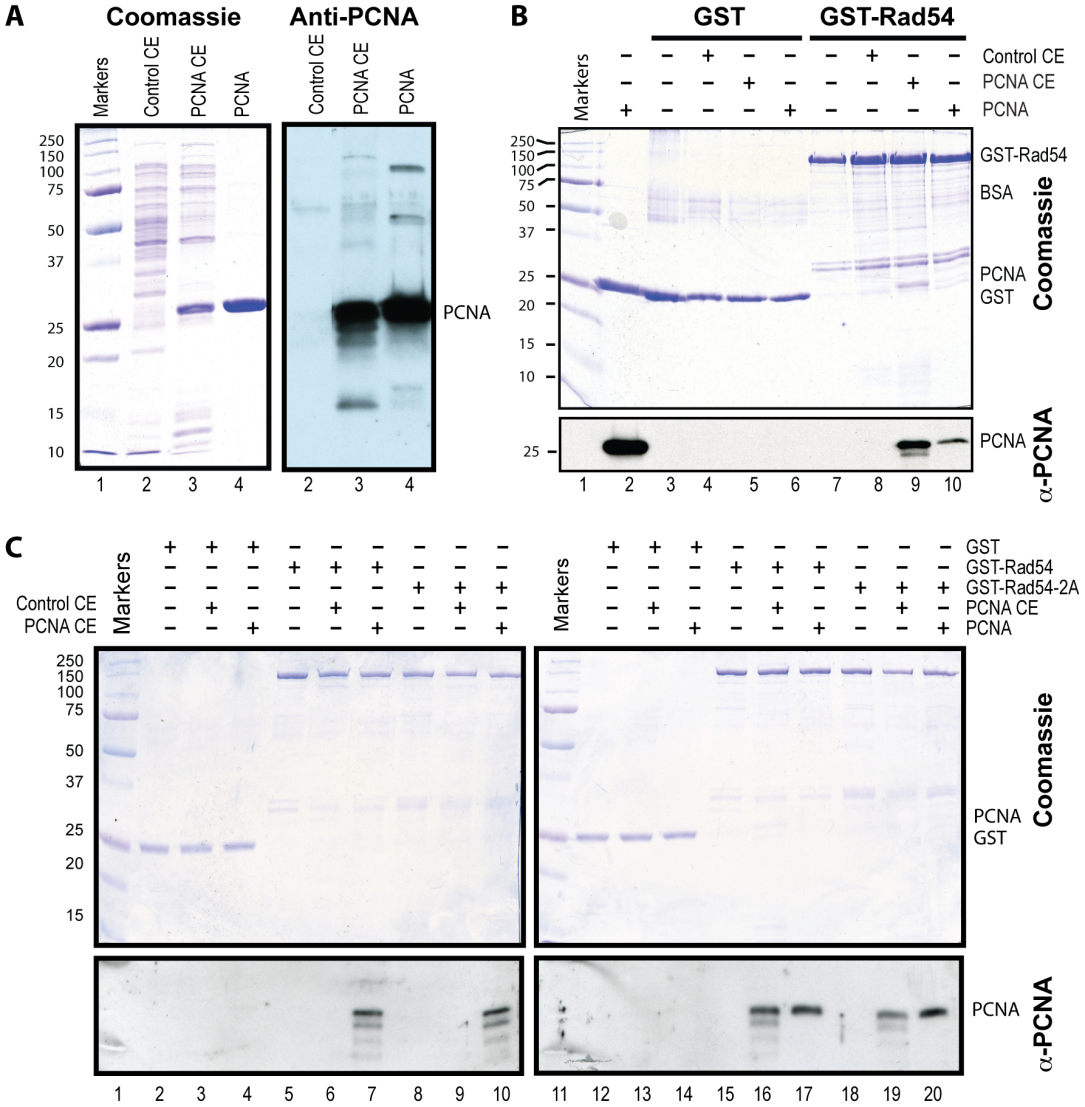
*In vitro* interaction between Rad54 and PCNA. **A.** Bacterial cell extracts and purified PCNA used in the GST pulldown assay. Lane 1, size markers; lane 2, 5 µg BL21(DE3) cell extract (Control CE); lane 3, 5 µg cell extract from BL21(DE3) overexpressing yeast PCNA (PCNA CE). PCNA was expressed significantly without IPTG induction. Lane 4 contains 2 µg GST-affinity purified PCNA. Left panel, Coomassie stained SDS-PAGE gel; right panel, immunoblots with anti-yeast PCNA anti-serum. **B.** Rad54 and PCNA (purified or in cell extract) interaction demonstrated by GST pulldown assays. Lane 1, size markers; lane 2, PCNA loading control (2 µg); lane 3–6, GST pulldown control; lane 7–10, GST-Rad54 pulldown assay. **C.** The Rad54-PCNA interaction does not depend on the canonical PIP box sequence. The interaction of GST-Rad54 and GST-Rad54-Y494A-F495A (GST-Rad54-2A) with purified PCNA and PCNA from cell extract was detected with GST pulldown assay visualized by both Coomassie (upper panel) and immunoblotting with yeast PCNA anti-serum (lower panel). Left panel, GST pulldown with cell extracts. Right panel, GST pulldown with PCNA cell extract (PCNA CE, 10 µg) and purified PCNA (2 µg). Lane 1 & 11, size markers; lane 2–4 & 12–14 were from GST (2 µg) pulldown control; lanes 5–7 & 15–17 were from GST-Rad54 (2 µg) pulldown, lanes 8–10 & 18–20 were from GST-Rad54-2A (2 µg) pulldown. Molecular weight markers are given in kDa.

To test whether Rad54 physically interacts with PCNA, we performed two-hybrid analysis and found that Rad54 interacts with PCNA in this assay (data not shown). To corroborate that this interaction is direct, we performed pulldown experiments with Rad54 using bacterial extracts containing PCNA or purified PCNA (Fig. 2). Rad54 co-precipitated PCNA from pure preparations and from PCNA-enriched bacterial extracts, but did not co-precipitate a negative control, GST (Fig. 2A, B). These experiments show that Rad54 directly interacts with PCNA.

PCNA interacts with the PIP box of an interacting protein *via* a hydrophobic pocket that docks the two key hydrophobic residues ([YF][YF]) in the consensus sequence [26], [48]. Mutating these key residues to alanine abrogates the interaction between the PIP box and PCNA and confirms PIP box-mediated interaction between PCNA and the candidate partner protein. We generated the corresponding mutant in Rad54 (Rad54-Y494A, F495A; here abbreviated as Rad54-2A) and purified the mutant protein from cells that lacked wild type Rad54 protein to avoid possible cross contamination. Surprisingly, there was no difference between mutant Rad54-2A and wild type Rad54 in their interaction with PCNA in the pull down assays with either bacterial extracts containing PCNA or purified PCNA (Fig. 2C) even under conditions of increased ionic strength to accentuate potential differences (Fig. S1 in File S1).

These data show that despite the presence of a conserved, canonical PIP box in Rad54, the Rad54-PCNA interaction occurred in a PIP box-independent fashion. To elucidate the function of the PIP box consensus sequence in Rad54 protein, we performed additional *in vivo* and *in vitro* experiments that are described below. We also analyzed further the Rad54-PCNA interaction (see Discussion).

### The PIP box consensus sequence is required for Rad54 *in vivo* function

**Figure 3:**
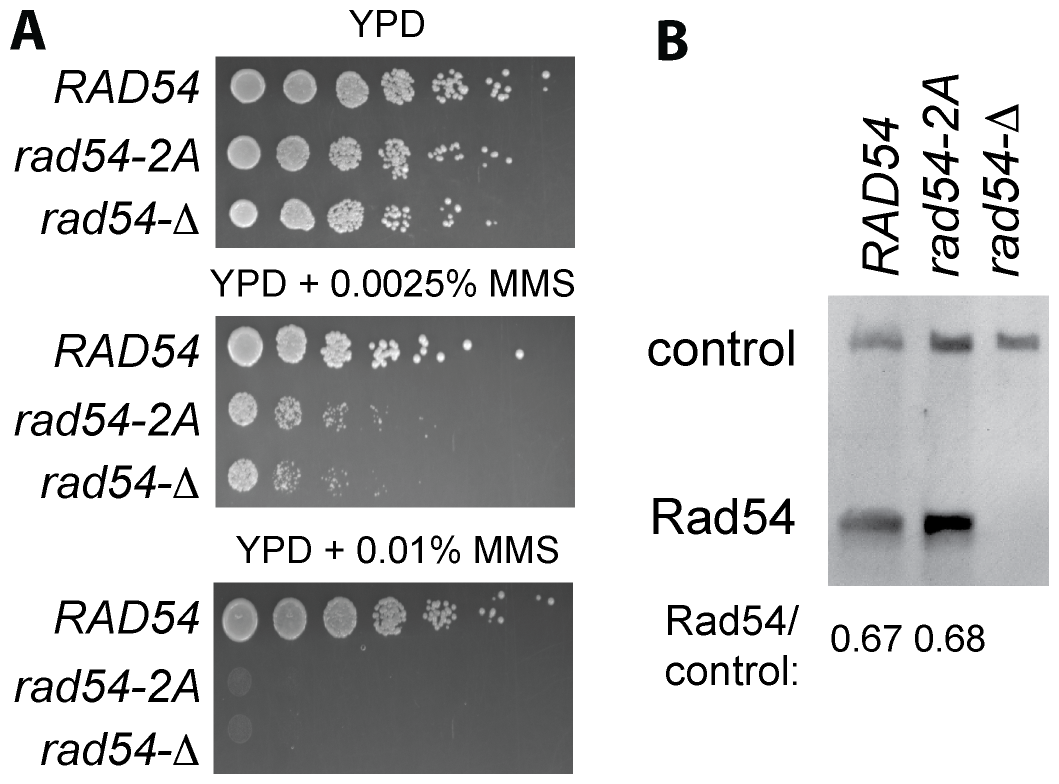
Rad54-2A is not functional *in vivo* and is expressed at normal protein levels. **A.**. *rad54-2A* cells are extremely MMS-sensitive. Cell cultures of wild type (WDHY2217), *rad54-Y494A, F495A ( = rad54-2A*, WDHY2625), and *rad54-Δ* (WDHY2571) were grown in YPD, diluted to OD_600_ of 1, followed by 6 five-fold serial dilutions. Cells were plated onto YPD containing 0.0025% or 0.01% MMS and grown at 30°C for 2 days and images were acquired to document cell growth. **B.** Comparison of endogenous wild type Rad54 and Rad54-2A protein levels. Extracts (100 µg) from wild type (WDHY2217), *rad54-2A* (WDHY2625) and *rad54-Δ* (WDHY2571) cells were immunoblotted with anti-*S. cerevisiae* Rad54 serum. The Rad54 protein levels were quantified using a crossreacting band as loading control. The ratios of the intensity of Rad54/control are indicated.

To query the functional significance of the PIP box consensus motif *in vivo*, we mutated the *RAD54* gene to generate a strain expressing the Rad54-2A protein from its native chromosomal locus. Rad54-deficient cells are highly sensitive to the alkylating agent methylmethane sulfonate (MMS) and cells expressing the Rad54-2A mutant protein (*rad54-2A*) were nearly as MMS-sensitive as the *rad54-Δ* null mutants (Fig. 3A). To exclude the possibility that the 2A mutations affect Rad54 protein stability, we established that the steady level of mutant Rad54-2A protein is identical to that of wild type Rad54, using a fortuitously cross-reacting band as a loading control in immunoblot analysis (Fig. 3B). Hence, the PIP box consensus motif is required for Rad54 function and defines a novel sequence element that is specifically conserved in the Rad54 subfamily and Snf2-family of SF2 helicases/translocases.

### The PIP box consensus sequence is required for Rad51-mediated D-loop formation

**Figure 4:**
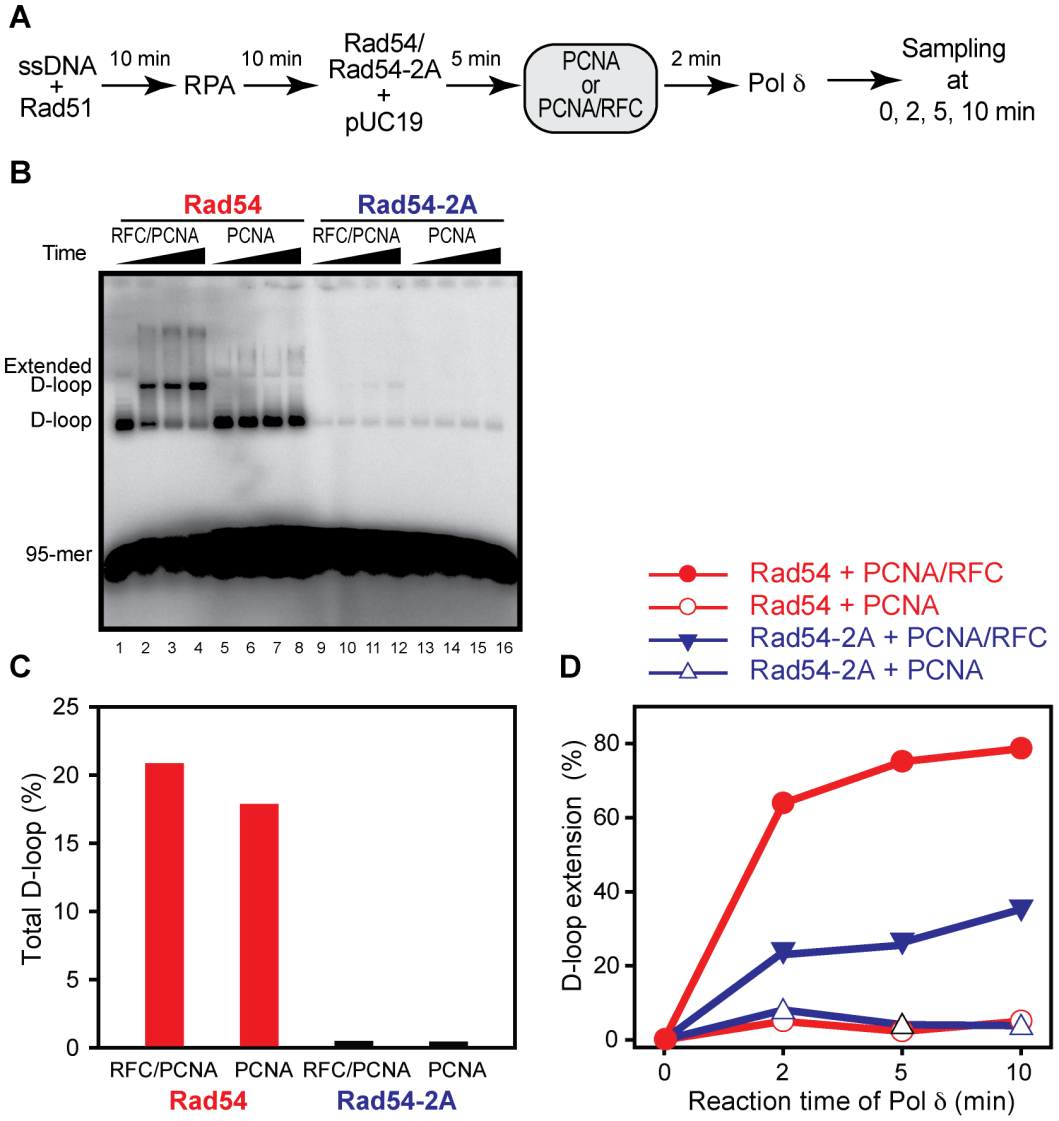
Rad54-2A is deficient in D-loop formation in reconstituted D-loop reactions containing PCNA. **A**, Reaction scheme of *in vitro* reconstitution system for Rad54-supported D-loop formation and D-loop extension by DNA polymerase δ. **B.** Analysis of D-loop formation and extension products in reactions with Rad54 (lanes 1–8) and Rad54-2A (lanes 9–16) by 1% native agarose gel electrophoresis in the presence of PCNA and RFC (lanes 1–4 and 9–12) or in the absence of PCNA (lanes 5–8 and 13–16). **C**. Quantification of reactions in *B* for total D-loops at 0 min DNA polymerase extension time. **D.** Quantification of D-loop extension as a function of time.

To understand the mechanistic basis of the *rad54*-2A *in vivo* defect, we reconstituted *in vitro* recombination reactions that combine D-loop formation by Rad51, Rad54, and RPA with D-loop extension by PCNA, RFC and DNA polymerase δ (Fig. 4A), as previously described [20]. In budding yeast, Rad54 is required for D-loop formation by Rad51 protein [49] and for extension of the invading strand in D-loop by DNA polymerases [30]. This assay is capable of monitoring separately D-loop formation (% total D-loops: extended+not extended D-loops) and D-loop extension (% extended D-loop of total D-loops) [20], [30]. These studies also showed that both DNA polymerase δ with PCNA/RFC and the Klenow fragment of *E. coli* DNA polymerase I extend the invading 3′-ends in D-loops even at very low D-loop concentrations. Wild type Rad54 supports robust D-loop formation (Fig. 4B, lanes 1 and 5) and efficient D-loop extension that is dependent on RFC-mediated loading of PCNA (Fig. 4B, compare lanes 2–4 with lanes 6–8). Rad54-2A, in contrast, was strongly impaired in D-loop formation (Fig. 4B lanes 9 and 13, Fig. 4C). The low D-loop levels formed in the Rad54-2A reactions were also less efficiently extended than in reactions containing wild type Rad54 (compare Fig. 4B lanes 2–4 with lanes 10–12, Fig. 4D). The low-level D-loop extension observed in reactions with Rad54-2A depended on PCNA loading by RFC (Fig. 4B, compare lanes 10–12 with lanes 14–16, Fig. 4D). These results demonstrate that the Rad54-2A mutant protein is deficient in Rad51-mediated D-loop formation. In addition, the data also suggest that Rad54-2A is impaired in ATPase-dependent removal of Rad51 from heteroduplex DNA, leading to less efficient extension by DNA polymerase, as inferred from our previous work that showed that Rad51 removal from heteroduplex DNA is required for the extension of the invading 3′-OH end by DNA polymerase [30].

**Figure 5:**
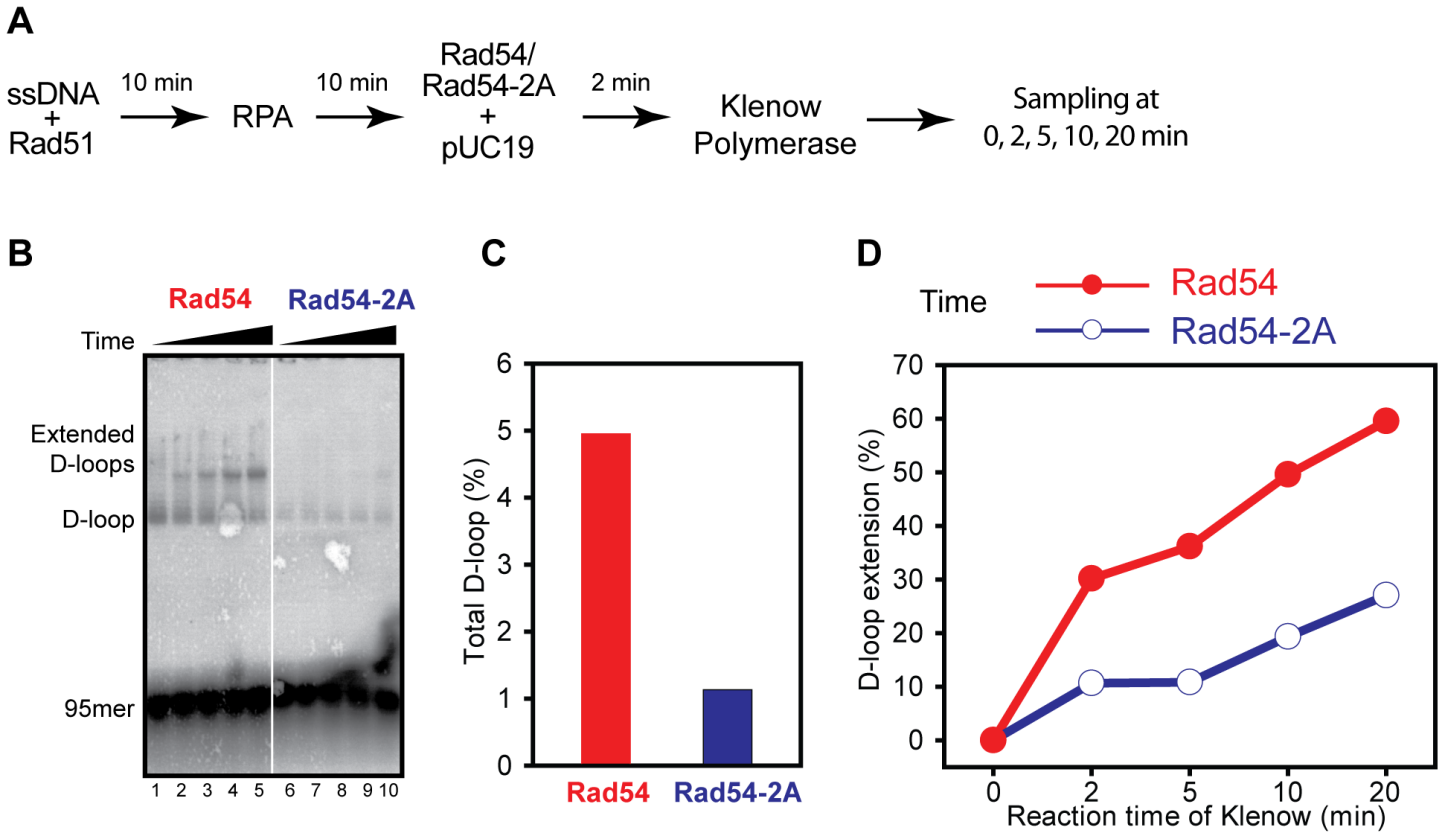
Rad54-2A is defective in D-loop extension catalyzed by Klenow polymerase. **A**. Reaction scheme of *in vitro* reconstitution system for Rad54-supported D-loop formation and D-loop extension by Klenow DNA polymerase. **B**. D-loop formation and extension by Klenow (lanes 1–10) in the presence of Rad54 (lanes 1–5) and Rad54-2A (lanes 6–10), respectively. **C**. Quantification of reactions in *B* for total D-loops at 0 min DNA polymerase extension time. **D.** Quantification of D-loop extension as a function of time.

To ascertain the above conclusion and to exclude any potential PCNA effect, we reconstituted D-loop extension assays using a PCNA-independent DNA polymerase in reactions lacking PCNA and RFC (Fig. 5). The Klenow fragment of *E. coli* DNA polymerase I efficiently extends Rad51-mediated D-loops in a Rad54-dependent fashion in the absence of PCNA/RFC [30]. As expected from our previous studies, wild type Rad54 protein supports this reaction, whereas the Rad54-2A mutant protein is highly deficient in D-loop formation. These results confirm the previous experiments including DNA polymerase δ with PCNA/RFC that Rad54-2A protein is defective in D-loop formation.

### The PIP box consensus sequence is required for Rad54 ATPase activity and dsDNA binding

**Figure 6:**
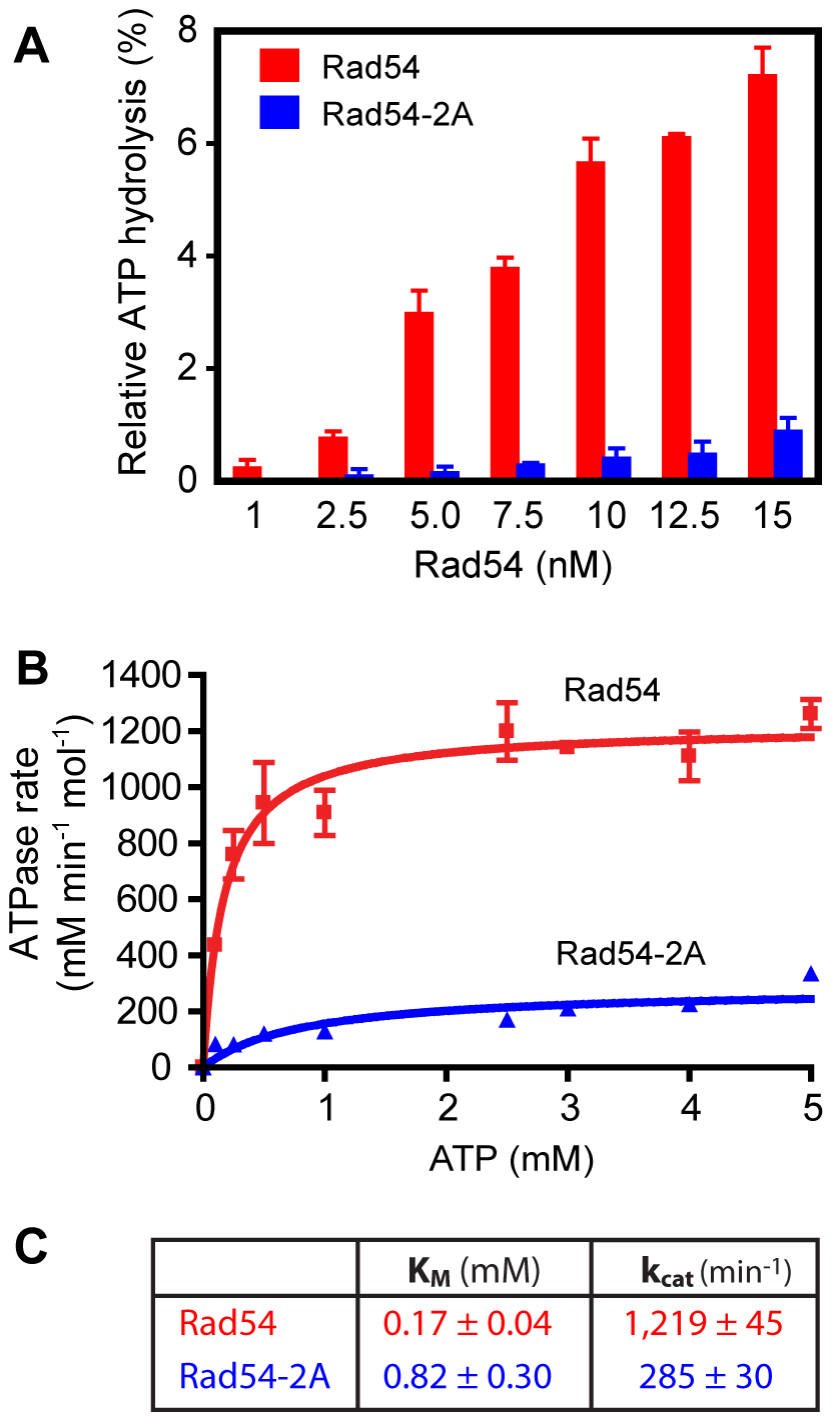
Rad54-2A exhibits reduced ATPase activity. **A**. The ATPase activities of Rad54 and Rad54-2A were analyzed in a protein titration using γ-^32^P[ATP]. **B.** Kinetic analysis of Rad54 and Rad54-2A ATPase activity. The R square values are: Rad54, 0.8995; Rad54-2A, 0.7632. **C.** Michaelis-Menten parameters of Rad54 and Rad54-2A as determined from the ATP titrations in B.

The ATPase activity of Rad54 is critical for its *in vivo* and *in vitro* functions [21], [22], and we tested using pUC19 supercoiled DNA whether the Rad54-2A deficiency in D-loop formation is caused by a defect in Rad54 ATPase activity. ATPase experiments using a protein titration revealed a strong defect in the Rad54-2A ATPase activity compared to the wild type Rad54 protein (Fig. 6A). Michaelis-Menten analysis (Fig. 6B) demonstrated that the ATPase defect of the mutant protein is explained by both reduced affinity for ATP and reduced ATP turnover, as Rad54-2A displayed a five-fold higher *K_M_* for ATP and a four-fold lower *k_cat_* (Fig. 6C).

**Figure 7:**
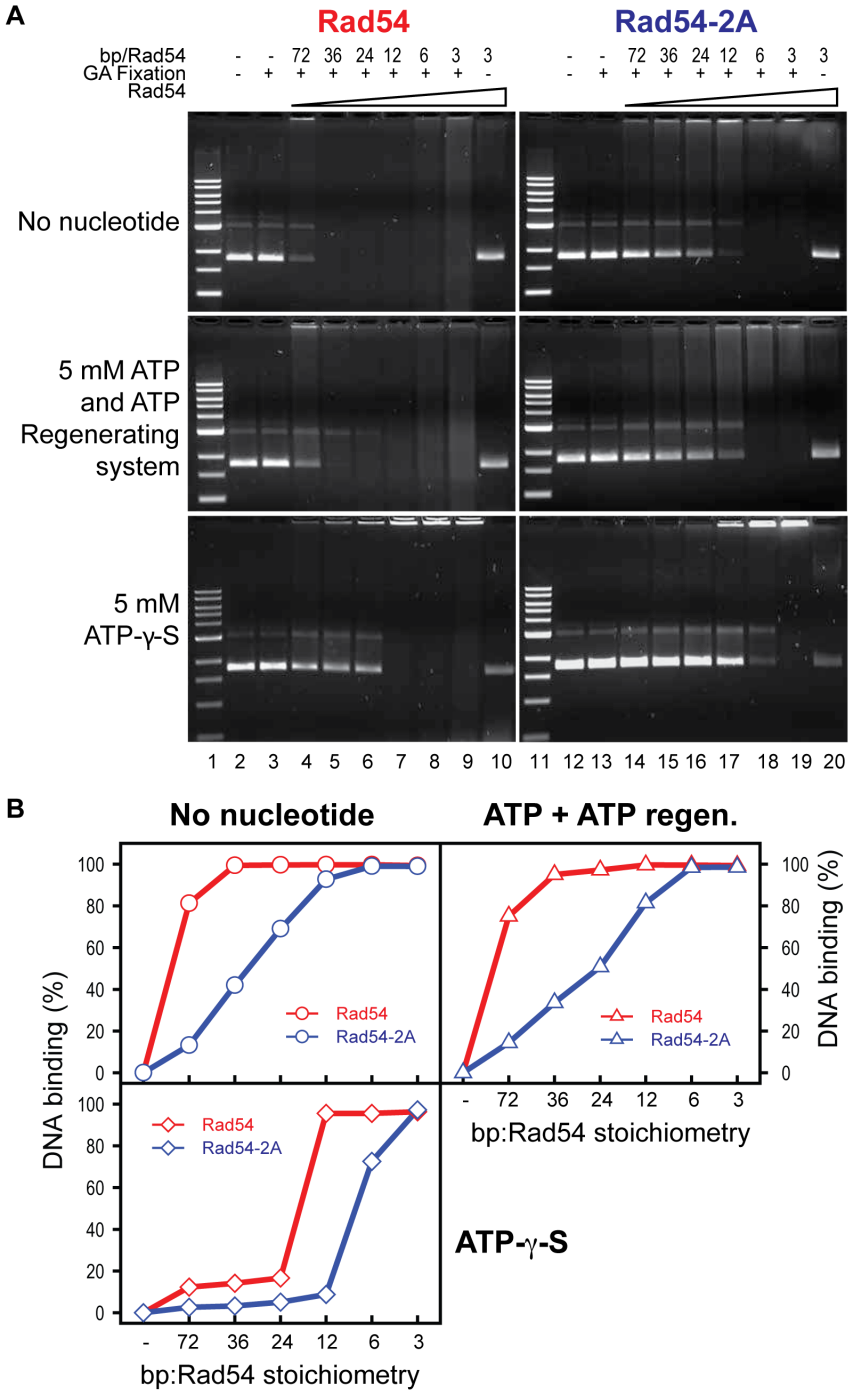
Rad54-2A exhibits a dsDNA binding defect. **A**. Rad54 (left panel) and Rad54-2A (right panel) were incubated with pUC19 supercoiled dsDNA at the indicated stoichiometries (bp DNA:Rad54 monomer) in the absence of nucleotide cofactor (upper panels), in the presence of 5 mM ATP and an ATP-regeneraton system (middle panels) or in the presence of 5 mM ATP-γ-S (lower panels) at 30°C for 15 minutes. The DNA-protein complexes were fixed by glutaraldehyde (GA) and visualized on a 1% agarose gel. **B**. Quantification of the DNA protein complexes from *A*.

Because the ATPase activity of Rad54 is dependent on dsDNA, we further tested whether the ATPase defect could be explained by an effect of the PIP box consensus sequence on Rad54 DNA binding [49], [50]. Binding of Rad54 to dsDNA can be monitored directly by a gel mobility shift and requires glutaraldehyde fixation to stabilize the Rad54-DNA complex [51]–[53] (see also Fig. 7). Wild type Rad54 binds to dsDNA independent of nucleotide cofactor hydrolysis in the presence of ATP or its slowly hydrolysable analog ATP-γ-S [51]–[53] (see also Fig. 7). To eliminate differences between the wild type Rad54 and the Rad54-2A mutant protein related to their different ATPase activities (Fig. 6) we conducted experiments with ATP in the presence of an ATP regeneration system and experiments in the absence of nucleotide co-factor. In the presence of ATP plus ATP regeneration system or in the absence of nucleotide cofactor, all available DNA is bound by wild type Rad54 at a stoichiometry of 36 bp per Rad54, whereas the Rad54-2A mutant protein requires a higher protein:DNA ratio and results in complete binding only at a ratio of 6–12 bp per Rad54 (Fig. 7A). The dsDNA binding defect of the Rad54-2A protein is also visible in the presence of ATP-γ-S (Fig. 7B), but under these conditions the protein:DNA ratios required for binding are different. These data suggest that the ATPase defect of the Rad54-2A mutant protein is related to a dsDNA binding defect. Since the stoichiometry for maximal binding was elevated with the Rad54-2A mutant protein, we tested whether overexpression of the Rad54-2A mutant could rescue some function *in vivo*. However, under various overexpression conditions and using several doses of MMS we did not observe any improvement in the DNA damage resistance (Fig. S3 in File S1).

### Further mutational analysis of the Rad54 PIP box consensus sequence

We generated additional mutations in the Rad54 PIP box consensus sequence (Table S3 in File S1) and analyzed the resulting alleles in a drop dilution assay to gauge plasmid-dependent complementation of *rad54*-Δ MMS sensitivity. For a subset of these alleles, we purified the mutant proteins and analyzed their ATPase activity (Table S3 in File S1). Our analysis allowed the following conclusions: 1) Alanine substitutions that in other studies disable PIP box function (L491A; Y494A,F495A; L491A,Y494A,F495A; QLYF-4A) virtually eliminate Rad54 function *in vivo* likely by affecting the ATPase activity; 2) Substitutions of the final two hydrophobic residues, Y494 and F495, of the PIP box consensus sequence show a pattern of *in vivo* function that does not suggest that these hydrophobic residues are involved in protein interactions, as substitutions that should affect protein interactions for their size (W, M) or charge (D, H, K) had inconsistent effects. Overall these data support our conclusion that the Rad54 PIP box consensus sequence is unlikely to represent a PCNA interaction motif but is rather an extension of a conserved ATPase motif that is critical for dsDNA binding and ATPase activity.

## Discussion

In this study we identified a perfect match to the PIP box consensus sequence in the Rad54 protein that is similarly present in its close paralogs Rdh54/Tid1 and human RAD54B, which also function in HR. In most PCNA-interacting proteins the PIP box is located in an unstructured linker at the N- or C-termini [26], [54]. Instead, the putative PIP box of Rad54 (and Rdh54 or RAD54B) is located in the middle of the protein contiguous with a critical motif of the ATPase catalytic core. However, exceptions exist, and some PIP boxes have been identified in central regions of a PCNA interaction partner such as the chromatin assembly factor subunit CAF-1 and DNA epsilon, where mutagenesis confirmed PIP box-dependent PCNA interaction [55]–[57]. Hence, it appeared possible that the internal PIP box consensus sequence of Rad54, and by extension yeast Rdh54 and human RAD54B, mediated PCNA interaction. Consistent with the presence of the PIP box consensus sequence we found that Rad54 interacts with PCNA. However, the PCNA interaction was independent of the PIP box consensus sequence, demonstrating that this sequence motif was not involved in PCNA interaction. Overexpression of a partner protein often can overcome an interaction defect (high copy suppression), but neither overexpression of PCNA (Fig. S4 in File S1) nor overexpression of Rad54-2A mitigated the extreme phenotype of the *rad54-2A* mutant (Fig. S3 in File S1). These findings do not prove but are consistent with our overall interpretation that this sequence motif in Rad54 is not involved in PCNA interaction. This interpretation also rests on more extensive mutational analysis of the two terminal hydrophobic residues of the Rad54 PIP box consensus sequence (Table S3 in File S1). These findings caution that the appearance of a perfect match to the PIP box consensus motif, which consist of only four conserved residues with defined spacing (Fig. 1A), is not sufficient to infer that the motif is responsible for PCNA interaction [26].

To further understand the significance of the Rad54-PCNA interaction, we purified various Rad54 truncation proteins and tested their interaction with PCNA (Fig. S5 in File S1). From these studies we could not identify a single linear region that was required or sufficient for PCNA interaction, rather different regions were involved depending on the construct. It is possible that the PCNA interaction motif of Rad54 is non-linear. However, this appears unlikely as both known PCNA interaction motifs, the PIP box and APIM, are short, linear amino acid sequence stretches [27], [28]. We have shown that the PIP box consensus sequence of Rad54 is not involved in PCNA interaction (Fig. 2). The Rad54 region (amino acids 1–168, Fig. S5 in File S1) interacting with PCNA does not contain a match to the alternative APIM motif [28]. Presentation of unstructured hydrophobic residues in the truncated Rad54 constructs we tested may result in non-specific PCNA interaction, and similar observations have been made independently (P. Burgers, pers. comm.). In sum, while Rad54 interacts with PCNA *in vitro*, we cannot yet define the critical interaction surfaces, and the biological significance of the Rad54-PCNA interaction remains to be determined.

Nevertheless, the putative PIP box consensus sequence appears to contribute significantly to Rad54 function *in vitro* and *in* vivo. This sequence directly follows motif III of the ATPase domain in Rad54 (and Rdh54, RAD54B) (Fig. 1) representing a conserved extension of motif III in the Rad54 sub-family of Snf2-like SF2 DNA motor proteins [11]. Little is known about the specific function of motif III. Functional analysis in budding yeast Snf2 showed that residue W935 was essential for function and mutation of this residue to alanine (Snf2-W935A) reduced activity by 88% as measured by transcriptional induction activity [58]. W935 in Snf2 is in the analogous position of motif III as F495 in Rad54 (see Fig. 1C). In fact, the Rad54-F495W substitution fully retains *in vivo* function and ATPase activity (Table S3 in File S1), corroborating that this residue is compatible with function in this position. Since there is no suggestion that Snf2 interacts with PCNA, these results are consistent with our interpretation that F495 and the PIP box consensus sequence extend motif III of the Rad54 ATPase.

The importance of motif III for Rad54 function has been demonstrated by the reconstruction of human cancer-associated mutations in budding yeast Rad54 [59]. The G325R mutation in human Rad54 has been associated with human cancer [60] and corresponds to residue G484 in the budding yeast Rad54 protein [59] (Fig. 1C). In yeast, the *rad54-G484R* mutation phenocopies the gene deletion. The purified mutant protein bound dsDNA similar to the wild type protein but was deficient in dsDNA-dependent ATPase activity and consequently D-loop formation [59]. These data show that whereas the canonical motif III in Rad54 is required for ATPase activity but not for dsDNA binding, the motif III extension that is specific for Rad54 may be critical for coordinating ATP hydrolysis with dsDNA binding. This interpretation is supported by structural modeling. Two crystal structures for Rad54 are presently available. One is the *Sulfolobus solfataricus* core motor domain bound to dsDNA [61], revealing two tandem RecA-like domains featuring two Rad54-specific insertions of unknown function. The *Sulfolobus* enzyme does not contain the conserved motif III extension (see Fig. 1C). The C-terminal core domain is rotated ∼180 degrees from its canonical position. The significance of this conformational change is unclear, and it could be related to the ATP-binding and hydrolysis cycle or may represent a conformation associated with DNA loading [62]. The second structure was obtained with the core domain of the zebrafish Rad54 protein, which contains the conserved motif III extension (Fig. 1C), and represents the canonical open configuration [47]. We have docked a dsDNA fragment into the yeast Rad54 structure that was modeled after the experimental zebrafish structure and found that the extended motif III in Rad54 directly contacts DNA (Fig. S3 in File S1), which could explain the DNA binding defect of the Rad54-2A mutant (Fig. 7).

In sum, biochemical and genetic observations paired with structural modeling support the conclusion that a PIP box consensus motif in Rad54, rather than presenting a canonical site for interaction with PCNA, represents instead a Rad54-specific extension of the SF1/SF2 ATPase motif III. In the case of Rad54 and its orthologs, this motif III extension is important for dsDNA binding, likely linking dsDNA binding with ATP hydrolysis. Our data are entirely consistent with and complementary to a parallel study conducted independently in the Rothstein, Klein, Kreicji, and Lisby laboratories (see co-submitted manuscript).

## Supporting Information

File S1 **Three Supplementary Tables, five Supplementary Figures, a Supplementary Method section and Supplementary References.**
(PDF)

## References

[1] Hunter N (2007) Meiotic recombination. In: Aguilera A, Rothstein R, editors. Homologous Recombination. Berlin-Heidelberg: Springer-Verlag. pp. 381–441.

[2] Li X, Heyer WD (2008) Homologous recombination in DNA repair and DNA damage tolerance. Cell Res 18: 99–113. 18166982 10.1038/cr.2008.1 PMC3087377

[3] Heyer WD (2007) Biochemistry of eukaryotic homologous recombination. In: Aguilera A, Rothstein R, editors. Molecular Genetics of Recombination. Berlin-Heidelberg: Springer-Verlag. pp. 95–133. 10.1007/978-3-540-71021-9 PMC3087616 21552479

[4] Heyer WD, Li X, Rolfsmeier M, Zhang XP (2006) Rad54: the Swiss Army knife of homologous recombination? Nucleic Acids Res 34: 4115–4125. 16935872 10.1093/nar/gkl481 PMC1616967

[5] Kanaar R, Troelstra C, Swagemakers SMA, Essers J, Smit B, et al (1996) Human and mouse homologs of the *Saccharomyces cerevisiae RAD54* DNA repair gene: Evidence for functional conservation. Curr Biol 6: 828–838. 8805304 10.1016/s0960-9822(02)00606-1

[6] Essers J, Hendriks RW, Swagemakers SMA, Troelstra C, deWit J, et al (1997) Disruption of mouse *RAD54* reduces ionizing radiation resistance. Cell 89: 195–204. 9108475 10.1016/s0092-8674(00)80199-3

[7] Ceballos SJ, Heyer WD (2011) Functions of the Snf2/Swi2 family Rad54 motor protein in homologous recombination. BBA 1809: 509–523. 21704205 10.1016/j.bbagrm.2011.06.006 PMC3171615

[8] Shinohara M, Shita-Yamaguchi E, Buerstedde JM, Shinagawa H, Ogawa H, et al (1997) Characterization of the roles of the *Saccharomyces cerevisiae RAD54* gene and a homologue of *RAD54*, *RDH54*/*TID1*, in mitosis and meiosis. Genetics 147: 1545–1556. 9409820 10.1093/genetics/147.4.1545 PMC1208330

[9] Klein HL (1997) *RDH54*, a *RAD54* homologue in *Saccharomyces cerevisiae*, is required for mitotic diploid-specific recombination and repair and for meiosis. Genetics 147: 1533–1543. 9409819 10.1093/genetics/147.4.1533 PMC1208329

[10] Wesoly J, Agarwal S, Sigurdsson S, Bussen W, Van Komen S, et al (2006) Differential contributions of mammalian Rad54 paralogs to recombination, DNA damage repair, and meiosis. Mol Cell Biol 26: 976–989. 16428451 10.1128/MCB.26.3.976-989.2006 PMC1347043

[11] Flaus A, Martin DMA, Barton GJ, Owen-Hughes T (2006) Identification of multiple distinct Snf2 subfamilies with conserved structural motifs. Nucleic Acids Res 34: 2887–2905. 16738128 10.1093/nar/gkl295 PMC1474054

[12] Cote J, Quinn J, Workman JL, Peterson CL (1994) Stimulation of GAL4 derivative binding to nucleosomal DNA by the yeast SWI/SNF complex. Science 265: 53–60. 8016655 10.1126/science.8016655

[13] Rattray AJ, Symington LS (1995) Multiple pathways for homologous recombination in *Saccharomyces cerevisiae* . Genetics 139: 45–56. 7705645 10.1093/genetics/139.1.45 PMC1206342

[14] Schild D (1995) Suppression of a new allele of the yeast *RAD52* gene by overexpression of *RAD51*, mutations in *srs2* and *ccr4*, or mating-type heterozygosity. Genetics 140: 115–127. 7635279 10.1093/genetics/140.1.115 PMC1206541

[15] Tan TLR, Kanaar R, Wyman C (2003) Rad54, a Jack of all trades in homologous recombination. DNA Repair 2: 787–794. 12826279 10.1016/s1568-7864(03)00070-3

[16] Mazin AV, Mazina OM, Bugreev DV, Rossi MJ (2010) Rad54, the motor of homologous recombination. DNA Repair 9: 286–302. 20089461 10.1016/j.dnarep.2009.12.006 PMC2827677

[17] Mazin AV, Alexeev AA, Kowalczykowski SC (2003) A novel function of Rad54 protein - Stabilization of the Rad51 nucleoprotein filament. J Biol Chem 278: 14029–14036. 12566442 10.1074/jbc.M212779200

[18] Wolner B, Peterson CL (2005) ATP-dependent and ATP-independent roles for the Rad54 chromatin remodeling enzyme during recombinational repair of a DNA double strand break. J Biol Chem 280: 10855–10860. 15653683 10.1074/jbc.M414388200

[19] Agarwal S, van Cappellen WA, Guenole A, Eppink B, Linsen SEV, et al (2011) ATP-dependent and independent functions of Rad54 in genome maintenance. J Cell Biol 192: 735–750. 21357745 10.1083/jcb.201011025 PMC3051825

[20] Li X, Stith CM, Burgers PM, Heyer W-D (2009) PCNA is required for initiating recombination-associated DNA synthesis by DNA polymerase δ. Mol Cell 36: 704–713. 19941829 10.1016/j.molcel.2009.09.036 PMC2784891

[21] Clever B, Schmuckli-Maurer J, Sigrist M, Glassner B, Heyer W-D (1999) Specific negative effects resulting from elevated levels of the recombinational repair protein Rad54p in *Saccharomyces cerevisiae* . Yeast 15: 721–740. 10398342 10.1002/(SICI)1097-0061(19990630)15:9<721::AID-YEA414>3.0.CO;2-W

[22] Petukhova G, Van Komen S, Vergano S, Klein H, Sung P (1999) Yeast Rad54 promotes Rad51-dependent homologous DNA pairing via ATP hydrolysis-driven change in DNA double helix conformation. J Biol Chem 274: 29453–29462. 10506208 10.1074/jbc.274.41.29453

[23] Gorbalenya AE, Koonin EV (1993) Helicases - amino acid sequence comparisons and structure function relationships. Curr Opin Struct Biol 3: 419–429.

[24] Walker JE, Saraste M, Runswick MJ, Gay NJ (1982) Distantly related sequences in the a- and b-subunits of ATP synthase, myosin, kinases and other ATP-requiring enzymes and a common nucleotide binding fold. EMBO J 1: 945–951. 6329717 10.1002/j.1460-2075.1982.tb01276.x PMC553140

[25] Singleton MR, Dillingham MS, Wigley DB (2007) Structure and mechanism of helicases and nucleic acid translocases. Annu Rev Biochem 76: 23–50. 17506634 10.1146/annurev.biochem.76.052305.115300

[26] Moldovan GL, Pfander B, Jentsch S (2007) PCNA, the maestro of the replication fork. Cell 129: 665–679. 17512402 10.1016/j.cell.2007.05.003

[27] Warbrick E (1998) PCNA binding through a conserved motif. BioEssays 20: 195–199. 9631646 10.1002/(SICI)1521-1878(199803)20:3<195::AID-BIES2>3.0.CO;2-R

[28] Gilljam KM, Feyzi E, Aas PA, Sousa MML, Muller R, et al (2009) Identification of a novel, widespread, and functionally important PCNA-binding motif. J Cell Biol 186: 645–654. 19736315 10.1083/jcb.200903138 PMC2742182

[29] Solinger JA, Kiianitsa K, Heyer W-D (2002) Rad54, a Swi2/Snf2-like recombinational repair protein, disassembles Rad51:dsDNA filaments. Mol Cell 10: 1175–1188. 12453424 10.1016/s1097-2765(02)00743-8

[30] Li X, Heyer WD (2009) RAD54 controls access to the invading 3-OH end after RAD51-mediated DNA strand invasion in homologous recombination in *Saccharomyces cerevisiae* . Nucleic Acids Res 37: 638–646. 19074197 10.1093/nar/gkn980 PMC2632917

[31] Erdeniz N, Mortensen UH, Rothstein R (1997) Cloning-free PCR-based allele replacement methods. Genome Res 7: 1174–1183. 9414323 10.1101/gr.7.12.1174 PMC310678

[32] Solinger JA, Heyer W-D (2001) Rad54 protein stimulates the postsynaptic phase of Rad51 protein-mediated DNA strand exchange. Proc Natl Acad Sci USA 98: 8447–8453. 11459988 10.1073/pnas.121009898 PMC37456

[33] Edgar RC (2004) MUSCLE: multiple sequence alignment with high accuracy and high throughput. Nucleic Acids Res 32: 1792–1797. 15034147 10.1093/nar/gkh340 PMC390337

[34] Waterhouse AM, Procter JB, Martin DM, Clamp M, Barton GJ (2009) Jalview Version 2–a multiple sequence alignment editor and analysis workbench. Bioinformatics 25: 1189–1191. 19151095 10.1093/bioinformatics/btp033 PMC2672624

[35] Thompson JD, Gibson TJ, Higgins DG (2002) Multiple sequence alignment using ClustalW and ClustalX. Curr Protoc Bioinformatics Chapter 2 Unit 2 3. 10.1002/0471250953.bi0203s00 18792934

[36] Eswar N, Webb B, Marti-Renom MA, Madhusudhan MS, Eramian D, et al (2006) Comparative protein structure modeling using Modeller. Curr Protoc Bioinformatics Chapter 5 Unit 5 6. 10.1002/0471250953.bi0506s15 PMC4186674 18428767

[37] Zhang XP, Galkin VE, Yu X, Egelman EH, Heyer WD (2009) Loop 2 in Saccharomyces cerevisiae Rad51 protein regulates filament formation and ATPase activity. Nucleic Acids Res 37: 158–171. 19033358 10.1093/nar/gkn914 PMC2615628

[38] Gallagher S, Winston SE, Fuller SA, Hurrell JGR (2008) Immunoblotting and immunodetection. Current Protoc Molecular Biology 10.18.11–10.18.28. 10.1002/0471142727.mb1008s83 18633991

[39] Kiianitsa K, Solinger JA, Heyer W-D (2003) NADH-coupled microplate photometric assay for kinetic studies of ATP-hydrolyzing enzymes with low and high specific activities. Anal Biochem 321: 266–271. 14511695 10.1016/s0003-2697(03)00461-5

[40] Zhang XP, Lee KI, Solinger JA, Kiianitsa K, Heyer WD (2005) Gly-103 in the N-terminal domain of Saccharomyces cerevisiae Rad51 protein is critical for DNA binding. J Biol Chem 280: 26303–26311. 15908697 10.1074/jbc.M503244200

[41] Pâques F, Haber JE (1997) Two pathways for removal of nonhomologous DNA ends during double-strand break repair in *Saccharomyces cerevisiae* . Mol Cell Biol 17: 6765–6771. 9343441 10.1128/mcb.17.11.6765 PMC232531

[42] Maloisel L, Bhargava J, Roeder GS (2004) A role for DNA polymerase delta in gene conversion and crossing over during meiosis in *Saccharomyces cerevisiae* . Genetics 167: 1133–1142. 15280229 10.1534/genetics.104.026260 PMC1470953

[43] Maloisel L, Fabre F, Gangloff S (2008) DNA polymerase delta is preferentially recruited during homologous recombination to promote heteroduplex DNA extension. Molecular and Cellular Biology 28: 1373–1382. 18086882 10.1128/MCB.01651-07 PMC2258756

[44] Wang XA, Ira G, Tercero JA, Holmes AM, Diffley JFX, et al (2004) Role of DNA replication proteins in double-strand break-induced recombination in *Saccharomyces cerevisiae* . Mol Cell Biol 24: 6891–6899. 15282291 10.1128/MCB.24.16.6891-6899.2004 PMC479734

[45] Fabre F, Boulet A, Faye G (1991) Possible involvement of the yeast POLIII DNA polymerase in induced gene conversion. Mol Gen Genet 229: 353–356. 1944222 10.1007/BF00267455

[46] Sebesta M, Burkovics P, Haracska L, Krejci L (2011) Reconstitution of DNA repair synthesis *in vitro* and the role of polymerase and helicase activities. DNA Repair 10: 567–576. 21565563 10.1016/j.dnarep.2011.03.003 PMC3119790

[47] Thoma NH, Czyzewski BK, Alexeev AA, Mazin AV, Kowalczykowski SC, et al (2005) Structure of the SWI2/SNF2 chromatin-remodeling domain of eukaryotic Rad54. Nature Struct Mol Biol 12: 350–356. 15806108 10.1038/nsmb919

[48] Schmidt KH, Derry KL, Kolodner RD (2002) *Saccharomyces cerevisiae* RRM3, a 5′ to 3′ DNA helicase, physically interacts with proliferating cell nuclear antigen. J Biol Chem 277: 45331–45337. 12239216 10.1074/jbc.M207263200

[49] Petukhova G, Stratton S, Sung P (1998) Catalysis of homologous DNA pairing by yeast Rad51 and Rad54 proteins. Nature 393: 91–94. 9590697 10.1038/30037

[50] Swagemakers SMA, Essers J, deWit J, Hoeijmakers JHJ, Kanaar R (1998) The human Rad54 recombinational DNA repair protein is a double-stranded DNA-dependent ATPase. J Biol Chem 273: 28292–28297. 9774452 10.1074/jbc.273.43.28292

[51] Li X, Zhang XP, Solinger JA, Kiianitsa K, Yu X, et al (2007) Rad51 and Rad54 ATPase activities are both required to modulate Rad51-dsDNA filament dynamics. Nucleic Acids Res 35: 4124–4140. 17567608 10.1093/nar/gkm412 PMC1919488

[52] Kiianitsa K, Solinger JA, Heyer WD (2002) Rad54 protein exerts diverse modes of ATPase activity on duplex DNA partially and fully covered with Rad51 protein. J Biol Chem 277: 46205–46215. 12359723 10.1074/jbc.M207967200

[53] Kiianitsa K, Solinger JA, Heyer WD (2006) Terminal association of Rad54 protein with the Rad51-dsDNA filament. Proc Natl Acad Sci USA 103: 9767–9772. 16785421 10.1073/pnas.0604240103 PMC1502528

[54] Shell SS, Putnam CD, Kolodner RD (2007) The N terminus of *Saccharomyces cerevisiale* Msh6 is an unstructured tether to PCNA. Mol Cell 26: 565–578. 17531814 10.1016/j.molcel.2007.04.024 PMC2001284

[55] Krawitz DC, Kama T, Kaufman PD (2002) Chromatin assembly factor I mutants defective for PCNA binding require Asf1/Hir proteins for silencing. Mol Cell Biol 22: 614–625. 11756556 10.1128/MCB.22.2.614-625.2002 PMC139734

[56] Moggs JG, Grandi P, Quivy JP, Jonsson ZO, Hubscher U, et al (2000) A CAF-1-PCNA-mediated chromatin assembly pathway triggered by sensing DNA damage. Mol Cell Biol 20: 1206–1218. 10648606 10.1128/mcb.20.4.1206-1218.2000 PMC85246

[57] Dua R, Levy DL, Li CM, Snow PM, Campbell JL (2002) In vivo reconstitution of *Saccharomyces cerevisiae* DNA polymerase epsilon in insect cells. Purification and characterization. J Biol Chem 277: 7889–7896. 11756442 10.1074/jbc.M108546200

[58] Richmond E, Peterson CL (1996) Functional analysis of the DNA-stimulated ATPase domain of yeast SW12/SNF2. Nucleic Acids Res 24: 3685–3692. 8871545 10.1093/nar/24.19.3685 PMC146154

[59] Smirnova M, Van Komen S, Sung P, Hannah LK (2004) Effects of tumor-associated mutations on Rad54 functions. J Biol Chem 279: 24081–24088. 15056673 10.1074/jbc.M402719200

[60] Matsuda M, Miyagawa K, Takahashi M, Fukuda T, Kataoka T, et al (1999) Mutations in the RAD54 recombination gene in primary cancers. Oncogene 18: 3427–3430. 10362365 10.1038/sj.onc.1202692

[61] Dürr H, Körner C, Müller M, Hickmann V, Hopfner KP (2005) X-Ray Structures of the *Sulfolobus solfataricus* SWI2/SNF2 ATPase Core and Its Complex with DNA. Cell 121: 363–373. 15882619 10.1016/j.cell.2005.03.026

[62] Durr H, Flaus A, Owen-Hughes T, Hopfner KP (2006) Snf2 family ATPases and DExx box helicases: differences and unifying concepts from high-resolution crystal structures. Nucleic Acids Res 34: 4160–4167. 16935875 10.1093/nar/gkl540 PMC1616948

