# Correction: A Conserved Sequence Extending Motif III of the Motor Domain in the Snf2-Family DNA Translocase Rad54 Is Critical for ATPase Activity

**DOI:** 10.1371/journal.pone.0101094

**Published:** 2014-06-23

**Authors:** The PLOS ONE Staff

A reference is omitted from the fifth sentence of the last paragraph in the Discussion section.

The sentence should read: Our data are entirely consistent with and complementary to a parallel study conducted independently in the Rothstein, Klein, Kreicji, and Lisby laboratories (Burgess et al., 2013).

The reference is: Burgess RC, Sebesta M, Sisakova A, Marini VP, Lisby M, et al. (2013) The PCNA Interaction Protein Box Sequence in Rad54 Is an Integral Part of Its ATPase Domain and Is Required for Efficient DNA Repair and Recombination. PLoS ONE 8(12): e82630. doi:10.1371/journal.pone.0082630
