# Supplementary material for: A Conserved Sequence Extending Motif III of the Motor Domain in the Snf2-Family DNA Translocase Rad54 Is Critical for ATPase Activity
Source: PLoS One. 2013 Dec 16;8(12):e82184. doi: 10.1371/journal.pone.0082184 (PMC3864901; doi:10.1371/journal.pone.0082184)
Supplement: File S1 — Three Supplementary Tables, five Supplementary Figures, a Supplementary Method section and Supplementary References. (PDF) [file pone.0082184.s001.pdf]

SUPPLEMENT S1

A conserved sequence extending motif III of the motor domain in the Snf2-family DNA translocase Rad54 is critical for ATPase activity

Xiao-Ping Zhang[1], Ryan Janke[1], James Kingsley[1], Jerry Luo[1], Clare Fasching[1], Kirk T. Ehmsen[1], and Wolf-Dietrich Heyer[1,2,*]

[1] Department of Microbiology & Molecular Genetics, University of California, Davis, Davis, CA 95616-8665, USA

[2] Department of Molecular & Cellular Biology, University of California, Davis, Davis, CA 95616-8665, USA

* Corresponding author: E-mail: wdheyer@ucdavis.edu

3 Supplementary Tables

5 Supplementary Figures

Supplementary Method

Supplementary References

Table S1. ***Saccharomyces cerevisiae*** **strains**

Strain	Relevant genotype
WDHY2217[1]	*ura3::LoxP*
WDHY2571[1]	*ura3::LoxP rad54Δ::URA3*
WDHY2625[1]	*ura3::LoxP rad54-Y494A,F495A (=rad54-2A)*
WDHY668	*MATa ura3-52 trp1 leu2Δ1 his3Δ200 pep4::HIS3 prb1Δ1.6R can1 GAL*<br>*MATα ura3-52 trp1 leu2Δ1 his3Δ200 pep4::HIS3 prb1Δ1.6R can1 GAL*
WDHY2655	*MATa ura3-52 trp1 leu2-Δ1 his3-Δ200 pep4 ::HIS3 prb1-Δ1.6R can1 GAL rad54Δ::KANmx*

[1] These strains are isogenic derivatives of W303 *RAD5* with the common genotype *MATa ade2-1 can1-100 his3-11,15 leu2-3,112 trp1-1*.

Table S2. Oligonucleotides

oIWDH	Sequence (5' to 3')	Purpose*
693	GCGCGGCCATGGGCTCCCCTATAC TAGGTTATTGG	F primer for cloning GST-tag DNA from pWDH597 to construct pET14b-GST vector
694	GCGCGCGGATCCCGGGAATCTCGA GGGCCCTGGAATAG	R primer for cloning GST-tag DNA from pWDH597 to construct pET14b-GST vector
873	GCGCGCCCTCGAGATATGTTAGAAGCAA AATTTGAAGAAGCATCCC	F primer for cloning PCNA gene from pWDH716 and to construct pET14b-GST-PCNA
874	GCGCGCCCCGGGTATACAACTATATAGA TAATTTA	R primer for cloning PCNA gene from pWDH716 and to construct pET14b-GST-PCNA
863	CACCCATTCAAAACGATGCTTCCGA ATATTTTGCT	*rad54-L491A*, F
864	GAGCAAAATATTCGGAAGCATCGTT TTGAATGGGTG	*rad54-L491A*, R
867	CGATCTTTCCGAAGCTGCTGCTCTA CTGAGTTTTTC	*rad54-Y494A-F495A*, F
868	GAAAAACTCAGTAGAGCAGCAGCT TCGGAAAGATCG	*rad54-Y494A-F495A*, R
865	CCATTCAAAACGATGCTTCCGAAGC TGCTGCTCTACTGAG	*rad54-L49A-Y494A-F495A*, F
866	CTCAGTAGAGCAGCAGCTTCGGAA GCATCGTTTTGAATGG	*rad54-L49A-Y494A-F495A,* R
910	CTGGTACACCCATTGCAAACGATGCTTCCG	*rad54-QLYF-4A*, F. Use *rad54-L491A-Y494A-F495A* as template
911	CGGAAGCATCGTTTGCAATGGGTGTACCAG	*rad54-QLYF-4A*, R. Use rad54-L491A-Y494A-F495A as template

1497	CAAAACGATCTTTCCGAATTGTGGGCTCTACTGAGTTTTTCG	*rad54-Y494L-F495W*, F
1498	CGAAAAACTCAGTAGAGCCCACAATTCGGAAAGATCGTTTTG	*rad54-Y494L-F495W*, R
1462	CGATCTTTCCGAAGCTTTTGCTCTACTG	*rad54-Y494A*, F
1463	CAGTAGAGCAAAAGCTTCGGAAAGATCG	*rad54-Y494A*, R
1464	CGATCTTTCCGAAGATTTTGCTCTACTG	*rad54-Y494D*, F
1465	CAGTAGAGCAAAATCTTCGGAAAGATCG	*rad54-Y494D*, R
1466	CGATCTTTCCGAAATCTTTGCTCTACTGAG	*rad54-Y494I*, F
1467	CTCAGTAGAGCAAAGATTTCGGAAAGATCG	*rad54-Y494I*, R
1485	CGATCTTTCCGAATTCTTTGCTCTACTGAG	*rad54-Y494F*, F
1486	CTCAGTAGAGCAAAGAATTCGGAAAGATCG	*rad54-Y494F*, R
1468	CGATCTTTCCGAAAAGTTTGCTCTACTGAG	*rad54-Y494K,* F
1469	CTCAGTAGAGCAAACTTTTCGGAAAGATCG	*rad54-Y494K,* R
1487	CGATCTTTCCGAATTGTTTGCTCTACTGAG	*rad54-Y494L,* F
1488	CTCAGTAGAGCAAACAATTCGGAAAGATCG	*rad54-Y494L,* R
1489	CGATCTTTCCGAAATGTTTGCTCTACTGAG	*rad54-Y494M,* F
1490	CTCAGTAGAGCAAACATTTCGGAAAGATCG	*rad54-Y494M,* R
1470	CGATCTTTCCGAATCTTTTGCTCTACTGAG	*rad54-Y494S,* F
1471	CTCAGTAGAGCAAAAGATTCGGAAAGATCG	*rad54-Y494S,* R
1472	CTTTCCGAATATGCTGCTCTACTGAG	*rad54-F495A*, F
1473	CTCAGTAGAGCAGCATATTCGGAAAG	*rad54-F495A,* R
1453	GATCTTTCCGAATATGATGCTCTACTGAGTTTTTC	*rad54-F495D,* F
1454	GAAAAACTCAGTAGAGCATCATATTCGGAAAGATC	*rad54-F495A,* R
1495	CTTTCCGAATATCATGCTCTACTGAG	*rad54-F495H,* F
1496	CTCAGTAGAGCATGATATTCGGAAAG	*rad54-F495H,* R
1476	CTTTCCGAATATATTGCTCTACTGAG	*rad54-F495I,* F
1477	CTCAGTAGAGCAATATATTCGGAAAG	*rad54-F495I,* R
1478	CGATCTTTCCGAATATAAGGCTCTACTGAGTTTTTC	*rad54-F495K,* F
1479	GAAAAACTCAGTAGAGCCTTATATTCGGAAAGATCG	*rad54-F495K,* R
1491	GATCTTTCCGAATATTTGGCTCTACTGAGTTTTTC	*rad54-F495L,* F
1492	GAAAAACTCAGTAGAGCCAAATATTCGGAAAGATC	*rad54-F495L,* R
1474	CGATCTTTCCGAATATATGGCTCTACTGAGTTTTTC	*rad54-F495M,* F
1475	GAAAAACTCAGTAGAGCCATATATTCGGAAAGATCG	*rad54-F495M,* R
1480	CTTTCCGAATATTCTGCTCTACTGAG	*rad54-F495S,* F
1481	CTCAGTAGAGCAGAATATTCGGAAAG	*rad54-F495S,* R
1493	GATCTTTCCGAATATTGGGCTCTACTGAGTTTTTC	*rad54-F495W,* F
1494	GAAAAACTCAGTAGAGCCCAATATTCGGAAAGATC	*rad54-F495W,* R
1506	AATTCCAGCTGACCACCATGGCGAAGGCCAAACTCTTCTC	Rad54 ad A-160
1507	GATCCCCGGGAATTGCCATGCCTCTATGGTACCAGTCGATATG	Rad54 ad B
1508	GCAAGACGCAGATTACCAGACAG	RAD54 F 3-26
1509	GATTCCAAGTTGTCGCATCACC	RAD54 R 2596-2575
1510	CTTGACGTTCGTTCGACTGATGAGC	5’ int_KL URA3
1511	GAGCAATGAACCCAATAACGAAATC	3’ int_KL URA3
1512	CATGGTGGTCAGCTGGAATTCGATGATGTAGTTTCTGGTT	KL URA3_ad a
1513	CATGGCAATTCCCGGGGATCGTGATTCTGGGTAGAAGATCG	KL URA3_ad b
1514	GACCTGAGTGGTGCCATGTATGC	RAD54 +340F
1515	GACAGCGTGAGATTTTCTTGCC	RAD54 -320R

*: F: forward primer; R: reverse primer

oIWDH1506-1513: *rad54-Y494A,F495A* (WHDY2625) integration primers

oIWDH1514-1515: *rad54*Δ (WDHY2571) integration primers

Table S3. Rad54 point mutants tested in this study

rad54-Δ/plasmid	488<br>Q	489<br>N	490<br>D	491<br>L	492<br>S	493<br>E	494<br>Y	495<br>F	MMS<br>resistance	ATPase
Empty vector									+	-
RAD54									++++	++++
rad54-K341R									+	
rad54-L491A				A					+	
Y494A,F495A<br>(=*rad54-2A*)							A	A	+	+
rad54-L491A,Y494A,F495A				A			A	A	+	
rad54-QLYF-4A	A			A			A	A	+	
rad54-Y494L,F495W							L	W	+++++	
rad54-Y494A							A		++	
rad54-Y494D							D		+	
rad54-Y494I							I		++++	
rad54-Y494F							F		+++	
rad54-Y494K							K		++++	
rad54-Y494L							L		+++	
rad54-Y494M							M		+++	
rad54-Y494S							S		+	
rad54-F495A								A	+++	+
rad54-F495D								D	++	
rad54-F495H								H	++++	
rad54-F595I								I	+	

rad54-F495K								K	++++	#
rad54-F495L								L	+	
rad54-F495M								M	++++	
rad54-F495S								S	+	
rad54-F495W								W	+++++	++++

All plasmids were transformed into WDHY2655 (Table S1) and the MMS sensitivity of the transformants was tested by spot assays on MMS-containing plates. More "+" symbols indicate better growth under MMS condition or better ATPase activity of the purified proteins. Rad54-F495W has about 90-100% activity of wild type ATPase, Rad54-2A and Rad54-F495A displayed about 5-10% of wild type ATPase activity (see also Fig. 6A). #: This mutant protein precipitated during purification and could not be purified.

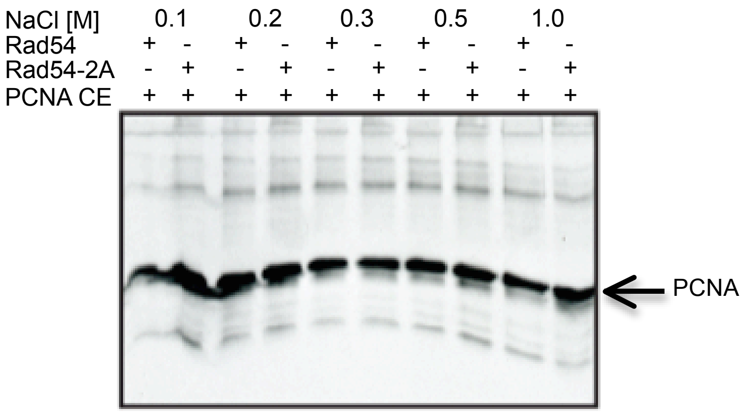

Figure S1. Rad54 wild type and Rad54-2A interaction with PCNA. The interaction of wild type Rad54 and Rad54-2A with PCNA was examined by pull down assays as described in Figure 2 and in Material and Methods with the final wash using buffer containing 0.1-1 M NaCl as indicated. As is evident from the intensity of the PCNA signal, wild type Rad54 and Rad54-2A show an equally strong salt-stable interaction with PCNA consistent with a hydrophobic interaction between both proteins. PCNA CE is bacterial cell extract containing yeast PCNA.

A. Zebrafish Rad54 with docked dsDNA

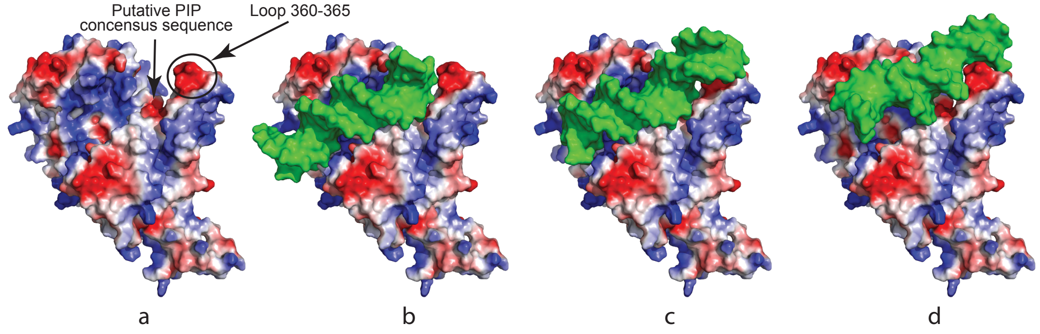

B. Yeast Rad54 with docked dsDNA

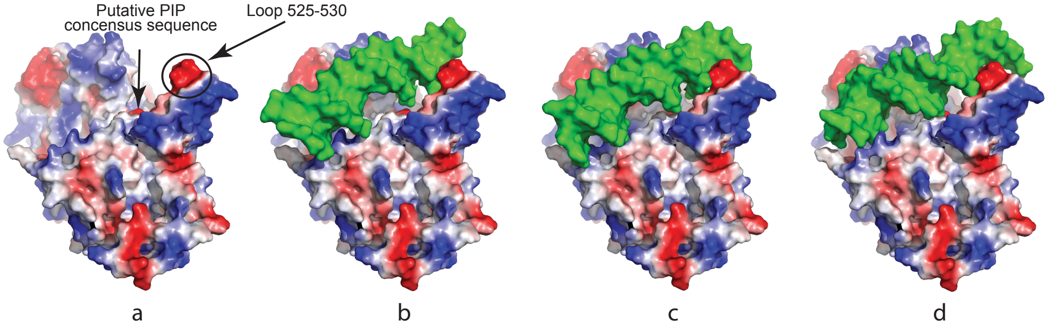

C. Calculated binding energy of the Rad54 and dsDNA

dsDNA binding mode	b	c	d
Zebrafish	-15.3	-15.3	-15.2
Yeast	-10.7	-12.1	-10.0

Figure S2. Modeling of dsDNA binding by Rad54. The Rad54 and dsDNA binding was studied with molecular docking technique as described in Supplementary Method. The dsDNA fragment preferentially binds to the cleft formed by the two RecA domains. Several binding modes (**b-d**) were identified by Autodock Vina, which mainly differ in the way that the dsDNA fragment contacts the Rad54 DNA binding cleft. The dsDNA fragment appears to rotate on its axis in the cleft and move forward. The putative PIP box sequence (labeled) is localized just under the DNA. **A**. dsDNA fragment was docked to zebrafish Rad54. **B**. The same dsDNA is docked to yeast Rad54 model. **C**. Calculated dsDNA-Rad54 binding energies in kcal/mol. The picture in **a** shows the surface of zebrafish (**A**) and yeast Rad54 (**B**) colored by “electrostatic potential”, blue means positive charges and red indicates negative charges. **b-c**, three potential binding states of dsDNA and Rad54.

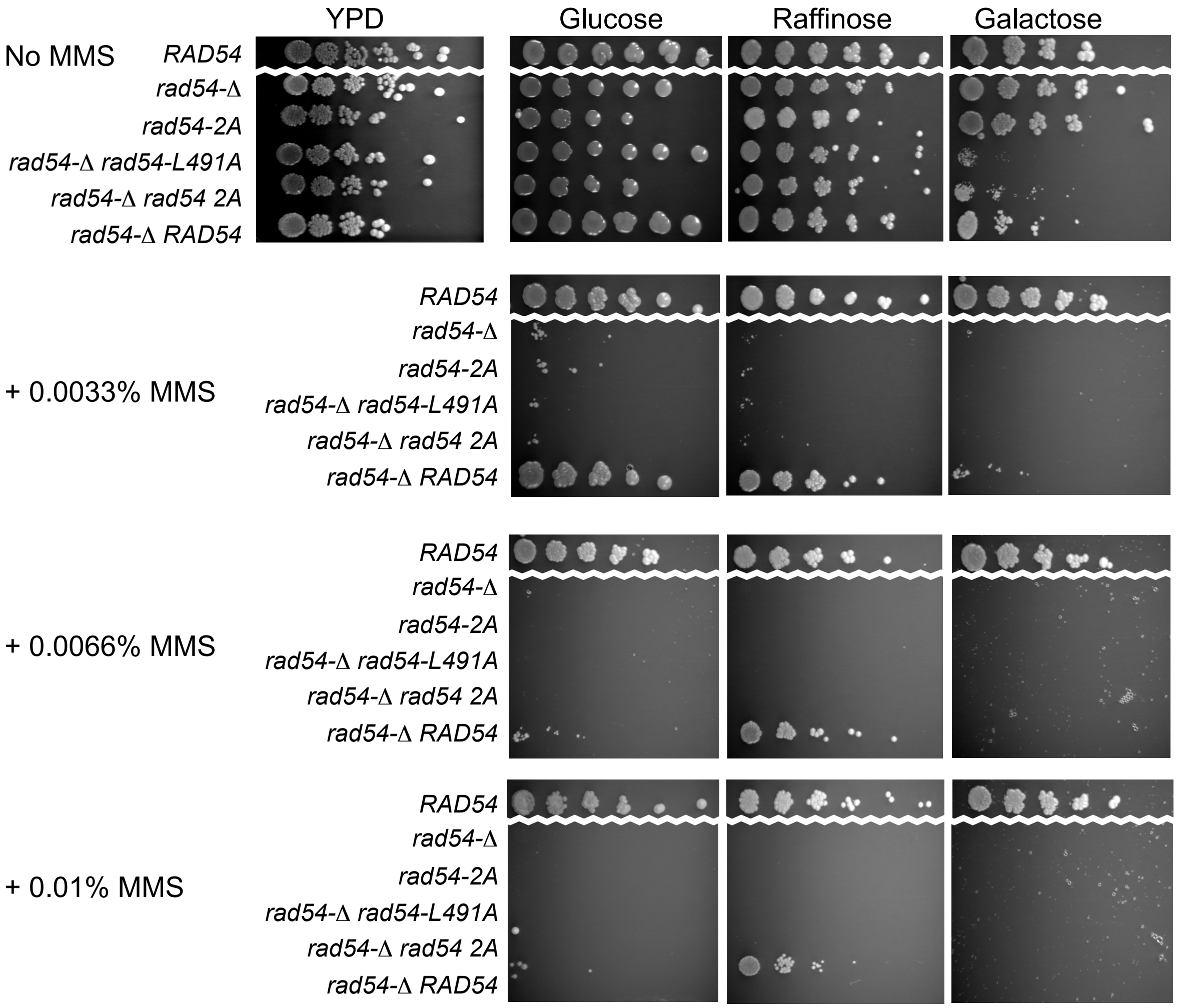

Figure S3. Rad54-2A overexpression does not rescue the *rad54-2A* defect. Cell cultures of wild type (WDHY2217), *rad54-Δ*(WDHY2571), and *rad54-Y494A, F495A (=rad54-2A*, WDHY2625) and *rad54-Δ* transformed with Rad54 overexpression plasmids where either wild type Rad54, Rad54-2A, or Rad54-L491A were expressed from the *GAL1-10* promoter. This particular G*AL1-10* overexpression vector shows leaky expression in glucose conditions, significant expression in raffinose, and strong overexpression in galactose. Cell cultures were adjusted to OD_{600} of 1, followed by six 5-fold serial dilutions and plating onto media containing the indicated concentration of MMS. Cells were grown at 30°C for up to 4 days and images were acquired to document cell growth. White zig-zag lines indicate where dilution series of strains not related to this figure have been removed. The data show that wild type Rad54 but

not Rad54-2A or Rad54-L491A can complement the MMS sensitivity caused by the *rad54-Δ* mutation. The complementation is most evident under conditions of low (glucose) or modest (raffinose) overexpression as strong overexpression of wild type Rad54 is known to elicit negative effects [1].

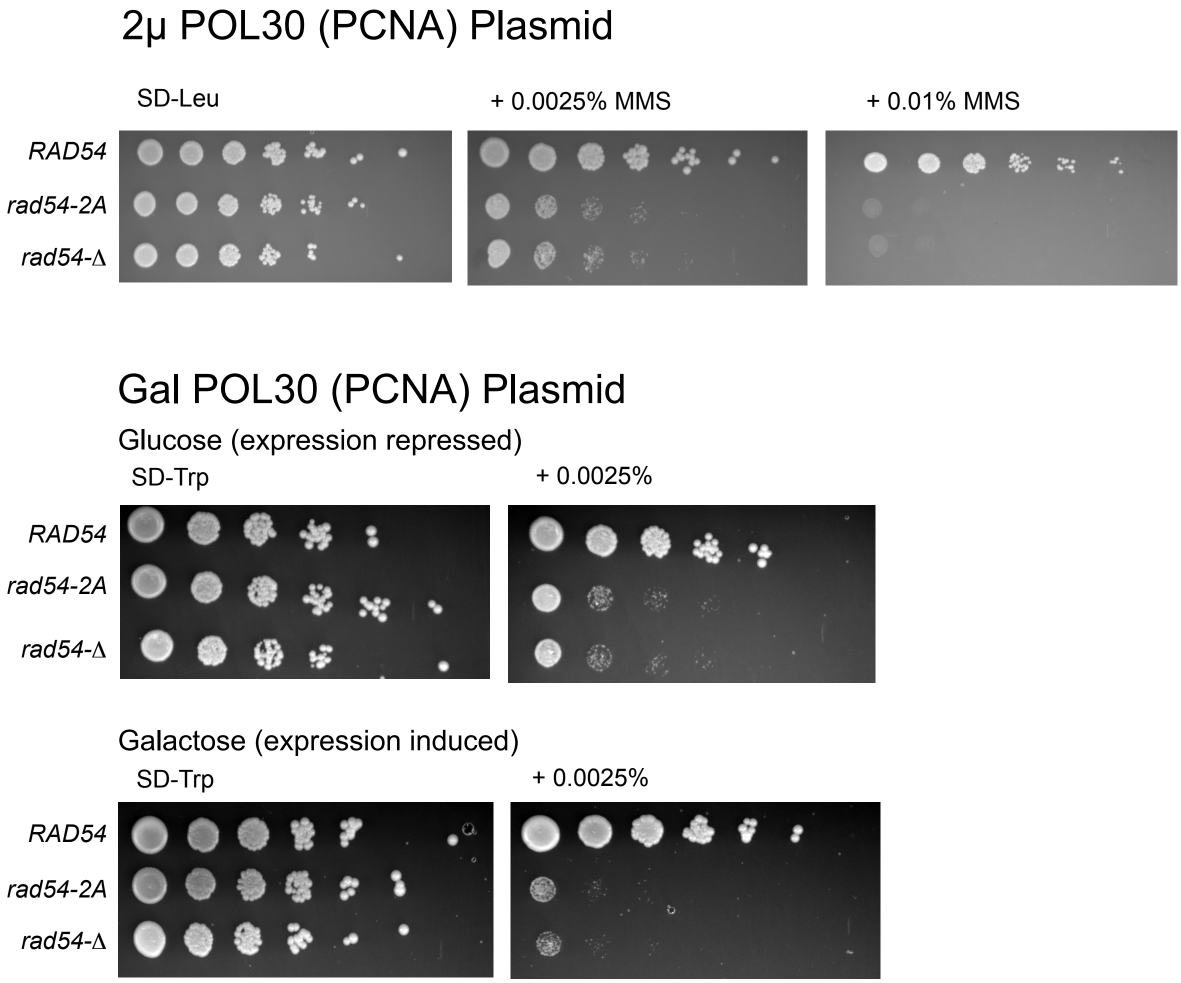

Figure S4. PCNA overexpression does not rescue the *rad54-2A* defect. Cell cultures of wild type (WDHY2217), *rad54-Y494A,F495A (=rad54-2A,* WDHY2625) and *rad54-*Δ were transformed with the indicated plasmids from which PCNA overexpression was either constitutive (2-micron *POL30* plasmid; pWDH769) or galactose inducible (*POL30* expression under control of the *GAL1-10* promoter; pWDH770). Overnight cell cultures were adjusted to OD_{600} of 1, followed by 6 five-fold serial dilutions and plating onto media lacking the indicated amino acid in order to maintain the plasmids. Plates contained the indicated concentration of MMS and cells were grown at 30°C for up to 4 days while images were acquired to document cell growth. The data show that PCNA overexpression does not rescue the MMS sensitivity of the *rad54-2A* or the *rad54-*Δ mutant.

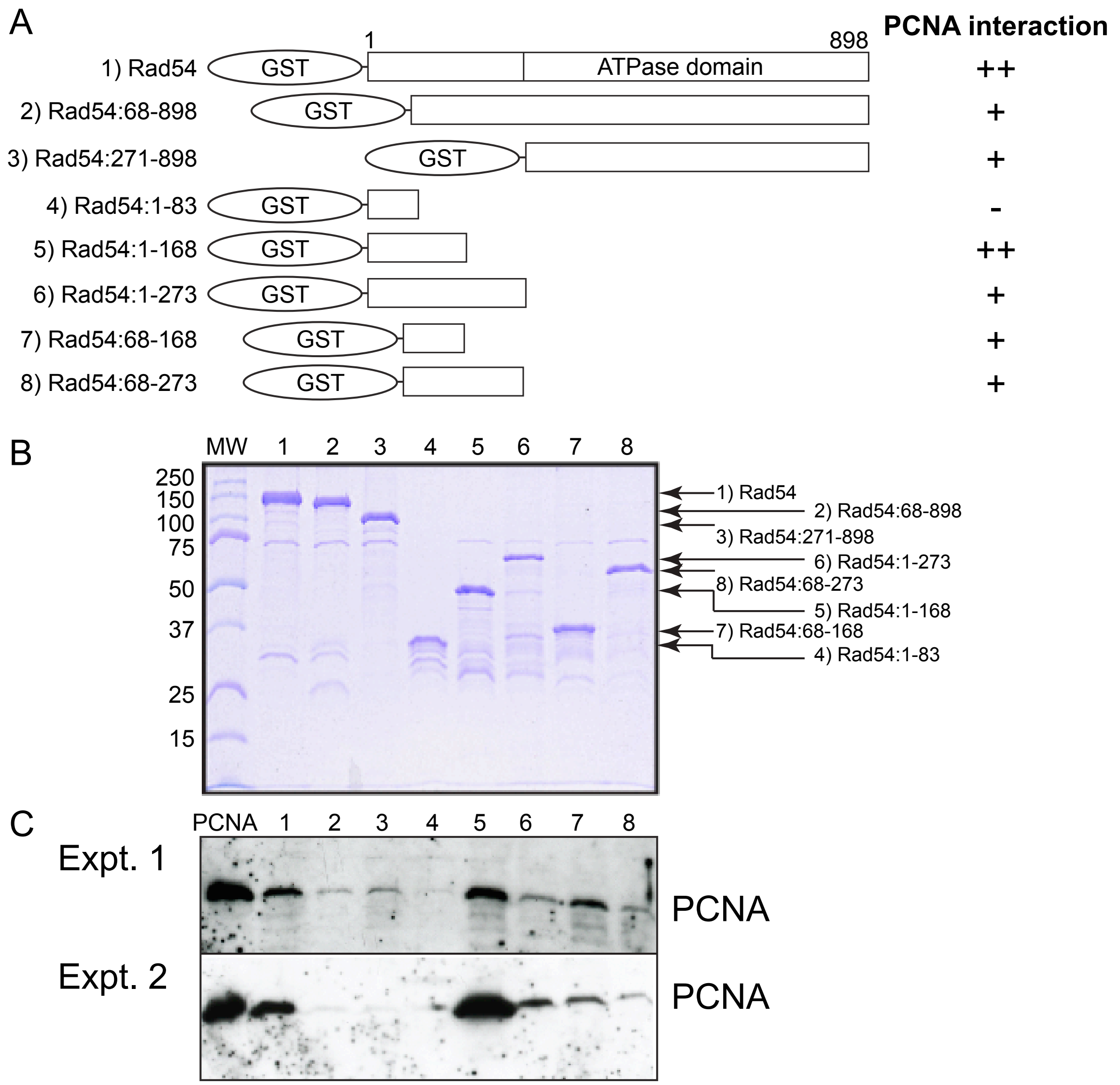

Figure S5. The Rad54 PCNA interaction site cannot be mapped to a single linear sequence region. A) Schematic of the full-length GST-Rad54 (898 amino acids) construct and the Rad54 truncation proteins studied and qualitative summary of the interaction data. The drawing is roughly to scale and the numbers indicate the Rad54 amino acids present in the construct. B) Coomassie-stained gel of partially purified Rad54 truncation proteins. The indicated Rad54 truncations labeled as in (A) were partially purified using glutathione-sepharose and SP-Sepharose [2]. MW: molecular weight markers in kDa. C) The interaction with PCNA was examined by pull down assays as described in Figure 2 and in Material and Methods.

Essentially the same results were found in two independent experiments and both are shown. The first lane in each blot is the PCNA loading control. Full-length Rad54 shows robust interaction with PCNA (see also Fig. S1). Deletion of the first 68 or 168 amino acids strongly diminishes interaction to the same extent. This would predict that the PCNA interaction domain is contained within the first 68 amino acids, but a fragment covering the first 83 amino acids shows no interaction. A fragment with the first 168 amino acids shows stronger interaction than the full-length protein, but a longer fragment with the first 273 amino acids displays strongly diminished interaction. Weak interaction is found with fragments encompassing amino acids 68-168 or 68-273.

Supplementary Method

The dsDNA and Rad54 binding was studied *in silico* with Autodock Vina 1.1.2 [3]. Yeast Rad54 structural model was generated through homology modeling as described in Materials and Methods. The crystal structures of Zebrafish Rad54 (1Z3I) [4] and *Sulfolobus solfataricus* SWI2/SNF2 ATPase and dsDNA (1Z63) [5] were retrieved from PDB database [6]. The double strand DNA fragment used for this study was extracted from the crystal structure 1Z63 (C and D chains). The C chain is 5'-ATTGCCGAAGACGAAAAAA-3' and its complementary strand, D chain is 5'-TTTTTTTCGTCTTCGGCAAT-3'. The dsDNA molecule was prepared in two steps: (1) Polar hydrogen and Gasteiger charges were added in Autodocktools-1.5.6rc package (http://mgltools.scripps.edu/). (2) dsDNA was generated as a docking ligand by running the command line python script "prepare_ligand4.py" from Autodocktools-1.5.6rc with -Z, -g and -l flags. The zebrafish and yeast Rad54 molecules were also prepared in Autodocktools-1.5.6rc with polar hydrogen and Kollman partial charges added. We treated both the dsDNA fragment and Rad54 as rigid bodies. With a grid center (25, 50, 20), the grid box (80, 64, 74) (zebrafish) and (80, 60, 72) (yeast) covers most of the zebrafish and yeast Rad54, including the cleft formed by the two RecA domains. The exhaustiveness and energy-range were set as 20 and 4, respectively. It took the Vina about 56 hours on an AMD Phenom II x6 1090T computer for each calculation. The results of the calculation were visualized and the images were generated in PyMol 1.5 (http://pymol.org/).

Supplementary References

1. Clever B, Schmuckli-Maurer J, Sigrist M, Glassner B, Heyer W-D (1999) Specific negative effects resulting from elevated levels of the recombinational repair protein Rad54p in *Saccharomyces cerevisiae*. Yeast 15: 721-740.
2. Kiianitsa K, Solinger JA, Heyer WD (2002) Rad54 protein exerts diverse modes of ATPase activity on duplex DNA partially and fully covered with Rad51 protein. Journal of Biological Chemistry 277: 46205-46215.
3. Trott O, Olson AJ (2010) AutoDock Vina: improving the speed and accuracy of docking with a new scoring function, efficient optimization, and multithreading. J Comput Chem 31: 455-461.

4. Thoma NH, Czyzewski BK, Alexeev AA, Mazin AV, Kowalczykowski SC, et al. (2005) Structure of the SWI2/SNF2 chromatin-remodeling domain of eukaryotic Rad54. Nature Struct Mol Biol 12: 350-356.
5. Dürr H, Körner C, Müller M, Hickmann V, Hopfner KP (2005) X-Ray Structures of the *Sulfolobus solfataricus* SWI2/SNF2 ATPase Core and Its Complex with DNA. Cell 121: 363-373.
6. Sussman JL, Lin D, Jiang J, Manning NO, Prilusky J, et al. (1998) Protein Data Bank (PDB): database of three-dimensional structural information of biological macromolecules. Acta Crystallogr D Biol Crystallogr 54: 1078-1084.
